# Paper-Based Electrochemical Device Modified with Palladium: Sensor for the Detection of Serotonin and an Immunosensor for the Detection of SOD1

**DOI:** 10.1021/acsomega.5c07375

**Published:** 2025-11-10

**Authors:** Jefferson H. S. Carvalho, Bruna S. Faria, Rafaela C. Freitas, Laís C. Brazaca, Bruno C. Janegitz

**Affiliations:** † 67828 Federal University of São Carlos (UFSCar), Araras 13604-900, São Paulo, Brazil; ‡ São Carlos Institute of Chemistry (IQSC), 28133 University of São Paulo (USP), São Carlos 13566-590, São Paulo, Brazil

## Abstract

The early and accurate detection of superoxide dismutase 1 (SOD1) and other biomarkers is crucial for the diagnosis and monitoring of neurodegenerative diseases such as amyotrophic lateral sclerosis. This study reports the development of a low-cost paper-based electrochemical sensor for the detection of serotonin (5-HT) and an immunosensor for the detection of SOD1, the potential biomarkers associated with these diseases. The sensor was fabricated using a conductive ink composed of carbon nanotubes and glass varnish onto an office paper substrate, with a palladium electrochemically deposited on the working electrode. To improve the device’s stability and water resistance, the paper surface was treated with beeswax, enhancing its hydrophobicity. Cyclic voltammetry was used to observe the electrochemical behavior, with differential pulse voltammetry applied to 5-HT. An analytical calibration curve was generated, with a limit of detection of 0.35 μmol/L for 5-HT, demonstrating a linear range of 7.00–100 μmol/L in PBS. The Pd-modified electrode enabled efficient immobilization of antibodies, facilitating the selective detection of SOD1 via antigen–antibody interactions. Electrochemical impedance spectroscopy was employed for label-free SOD1 quantification, yielding a linear response in the range of 1.0–100 nmol·L^–1^ and a limit of detection of 0.72 nmol·L^–1^. The proposed electrochemical immunosensor demonstrates high sensitivity, selectivity, and affordability, making it a promising tool for early stage screening of neurodegenerative disease biomarkers in real-world clinical samples.

## Graphical abstract

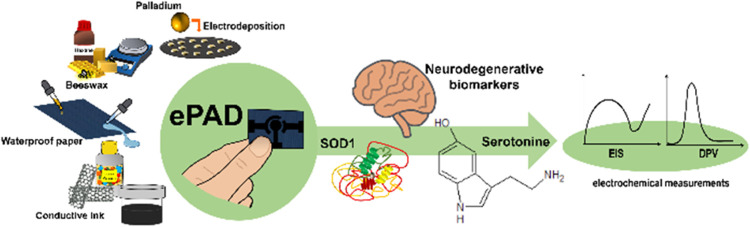

## 1 Introduction

In recent years, the demand for innovative and efficient analytical platforms has led to the development of paper-based electrochemical devices (ePADs). These devices have attracted attention for their ability to provide sensitive determinations while being easy to produce, low-cost, and well-suited for point-of-care applications.^1–4^ These devices are recognized and widely used in analytical applications due to their straightforward operation, which utilizes hydrophilic cellulose fibers in the structure, enabling hydrophilic liquids to enter and be transported solely through capillary forces.^5^

The increasing use of paper as a substrate for biosensors is largely due to its beneficial properties, including biodegradability, biocompatibility, accessibility from sustainable or recycled sources, portability, and low cost.^6,7^ The versatility of paper is enhanced by its variety of forms, such as filter paper, paperboard, office paper, and carbon fiber paper, each offering distinct characteristics for different applications.^8^ For optimal device performance, factors such as capillarity, hydrophilicity, and porosity must be considered depending on the specific requirements of the analytical application.^9^ While electrochemical measurements can be performed directly on paper substrates, for long-term applications or when repeated modifications to the electrodes are required, waterproofing is crucial to maintain device durability.^7,10^ Beeswax has emerged as a popular waterproofing agent due to its low cost, accessibility, inertness in biological applications, and effective waterproofing properties.^11^

In contrast to gold, which is often used in immunosensors,^12–14^ palladium (Pd) nanoparticles have gained attention as a cost-effective alternative.^15–18^ Pd offers several advantages, including lower cost, biocompatibility, higher signal generation capacity, and enhanced stabilization when used with carbon-based materials.^19^ These characteristics made Pd a key choice for the development of the immunosensor in this work.

Amyotrophic lateral sclerosis (ALS) is a debilitating neurodegenerative disease that leads to the progressive degeneration of motor neurons in the brain and spinal cord, affecting both upper and lower motor neurons.^20^ Early detection of ALS is critical, and identifying reliable biomarkers for the disease has become a key research focus. One of the most promising biomarkers associated with ALS is the copper/zinc ion-binding superoxide dismutase (SOD1).^20,21^

SOD1 is an important antioxidant enzyme that plays a critical role in managing oxidative stress in the body. It acts as the first line of defense against oxidative damage in both physiological and pathological conditions.^22^ Research has shown that the concentration of SOD1 varies significantly between ALS patients and healthy individuals. The latest study concerning SOD1 in ALS patients comes from Simonini et al.,^23^ who aimed to investigate a range of biomarkers that change for ALS-SOD1 patients while also noting that identifying specific changes for SOD1 still requires further investigation. Therefore, it is essential to stress that the device developed in this research is designed to function at concentrations reported in the literature and is proposed for use in samples whose concentrations still need further evaluation and alternative methods.

5-Hydroxytryptamine (5-HT), or serotonin, is a neurotransmitter derived from the amino acid tryptophan. It is crucial in various physiological processes, including mood regulation, sleep, and appetite control.^24^ Recently, 5-HT has gained attention as a potential biomarker for neurodegenerative diseases, particularly Parkinson’s disease and amyotrophic lateral sclerosis (ALS). Compared to healthy controls, reduced levels of 5-HT in affected individuals suggest its potential as a diagnostic marker.^25^

In summary, this work proposes an ePAD created through screen printing to produce a three-electrode electrochemical system using conductive carbon nanotube ink and stained glass varnish on a beeswax-sealed office paper substrate. The ePAD’s applications are divided into two parts: the first as an electrochemical sensor for detecting serotonin, a potential biomarker of neurodegenerative diseases. The ePAD evaluates the behavior of electrodeposited Pd nanoparticles on the working electrode surface and their potential to increase sensitivity. Subsequently, the ePAD is used as an electrochemical immunosensor for superoxide dismutase 1, another biomarker of neurodegenerative diseases. This demonstrates Pd as a metallic alternative to other metals already common in biosensing, while also offering a new, simple, and disposable electrochemical system.

## 2 Materials and Methods

### 2.1 Reagents and Solutions

For the construction of the electrochemical immunosensor, Pd nitrate (II), cysteamine (CYS), glutaraldehyde (GA), bovine serum albumin (BSA), and buffer solution reagents were obtained from Sigma-Aldrich and/or Fluka. Ultrapure water (resistivity ≥18.2 MΩ cm) was sourced from Millipor Synergy and utilized in all solution preparations. The conductive ink was prepared using the work of Carvalho et al.^26^ as a reference. An A4 blue screen paper (ref: 66668793 180g/m^2^) produced by CANSON was used as the substrate. In the electrochemical characterization, 0.1 mmol L^–1^ [Fe­(CN)_6_]^4–/3–^ was employed as a redox probe in 0.1 mol·L^–1^ KCl. A Pd solution was prepared from a superconcentrated Pd nitrate (II) solution in 0.1 mol·L^–1^ HNO3. This superconcentrated solution was diluted in 0.1 mol·L^–1^ HNO3 to yield a 1.0 mmol L^–1^ Pd solution. Phosphate buffer saline (PBS), pH was prepared from 0.1 PB (0.1 mol·L^–1^ Na_2_HPO_4_ and 0.1 mol·L^–1^ KH_2_PO_4_) with the addition of 0.1 mol·L^–1^ KCl and NaCl. CYS (10 mmol L^–1^) and GA (5 mmol L^–1^) were prepared in 0.1 mol·L^–1^ PBS, pH 8.6. AntiSOD1 was prepared in 1× saline tris buffer, and SOD1 in 0.1 mol·L^–1^ PBS, pH 7.5.

### 2.2 Equipment

All electrochemical measurements were performed using the Autolab PGSTAT101 and the VIONIC potentiostats/galvanostats (Metrohm, Eco Chemie). The measurements were monitored by the software NOVA 2.1.4 and INTELO 1.4, respectively. The pH values were measured with the Metrohm pH meter 827. Scanning electron microscopy (SEM) of the sensor and paper surface was conducted using a ThermoFisher Scientific Prisma E, applying a voltage of 10 and 20 kV in low vacuum mode at 50 Pa. To ensure the conductive ink was sheared and mixed, a double asymmetric centrifuge (SpeedMixerTM DAC 150.1 FVZ-K FlackTek Inc.) was used. Additionally, for the fabrication of the adhesive mask with the three-electrode system, a cutting printer (Silhouette Cameo 3) was employed.

### 2.3 Preparation of the Beeswax Solution and Waterproofing of the Paper Substrate

A beeswax solution was used to waterproof the paper substrate after observing that the paper could not support the solution for the entire duration of the electrochemical measurements (>10 min) (Figure S1). The work of de Oliveira et al.^11^ served as a reference for this procedure. In summary, a beeswax solution was prepared by dissolving 1 g of beeswax in 100 mL of hexane. The compounds were mixed for 2 min at 60 °C. After this, a paintbrush was used to apply the solution to the paper in three layers. The process is completed after 24 h of drying.

### 2.4 ePAD Fabrication

The ePAD fabrication was possible through the work of Carvalho et al.,^26^ who constructed a conductive ink based on carbon nanotubes and glass varnish, utilizing PET as a substrate. In this work, we replace the substrate with paper and adjust the sensor size. To reduce reagent and solution consumption, the electrode arrangement was modified to a similar disposable design used by Camargo et al.,^27^ but with smaller electrode dimensions. The sensor dimensions are shown in Figure S2, which compares the sensor with a U.S. quarter-dollar coin and a ruler. Additionally, the CNTs–GV/ePAD sensor measures 2 cm × 1 cm, with a geometric area of the working electrode equal to 0.132 cm^2^ and a volume of 40 μL for electrochemical measurements. Figure 1A presents a schematic of the CNTs–GV/ePAD. To better visualize the sensor production process, Video S1 was created to demonstrate each step. After sensor production, a modification to the working electrode surface was realized using a Pd solution. This step was necessary to construct the immunosensor.

**Figure 1:**
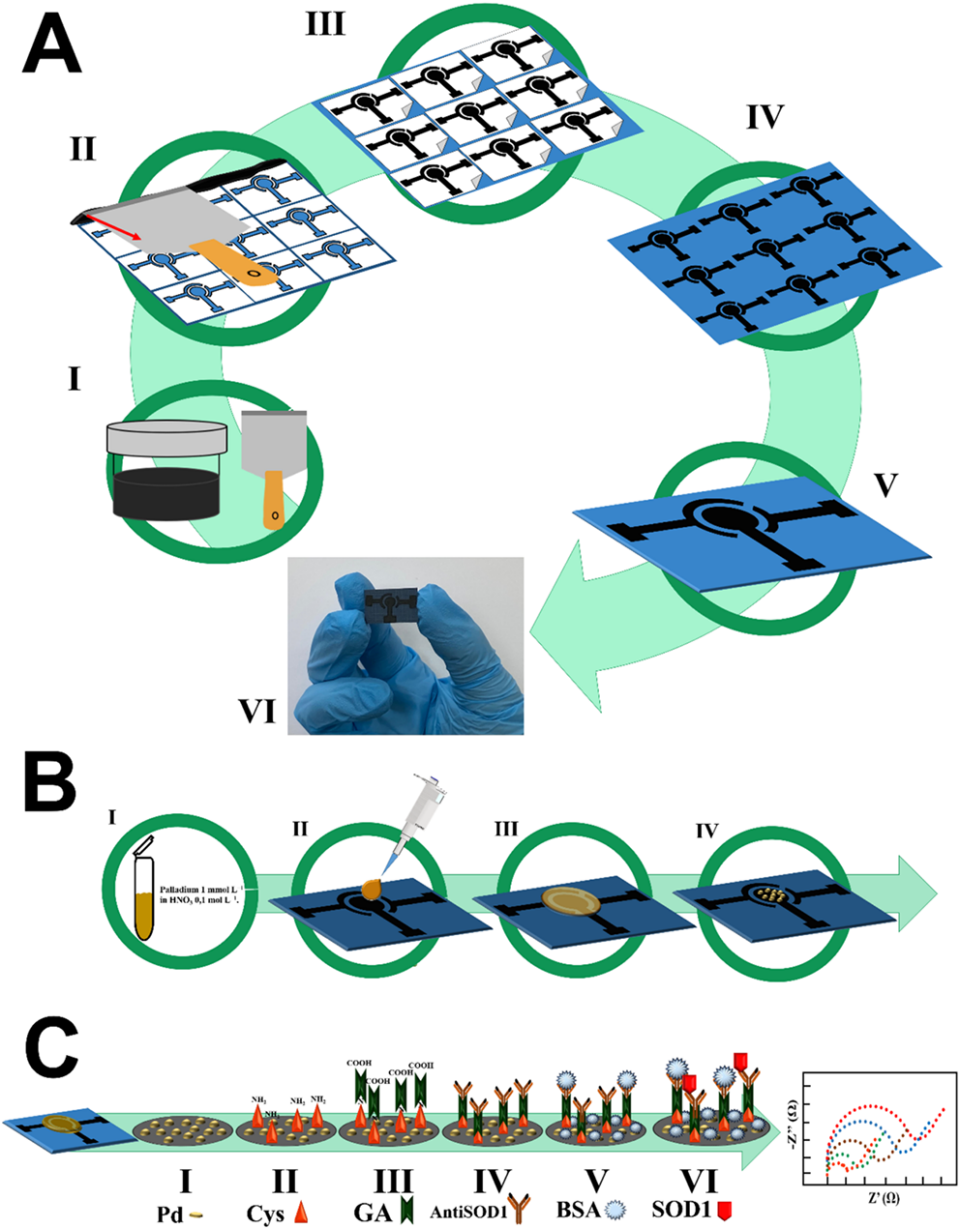
(A) Schematic representation of the electrochemical sensor preparation utilizing carbon nanotubes and glass varnish. I—Weighing of the conductive ink compounds and homogenization of the mixture by a centrifuge. II—Conductive ink spread on the adhesive-masked surface with the aid of a spatula. III—Removing the adhesive mask; IV—Drying of the electrochemical sensor for 24h, at room temperature; V—Cutting of units after drying; VI—Final picture of the electrochemical sensor. (B) Schematic representation of the electrodeposition of Pd using chronoamperometry. I—Pd nitrate solution was prepared in 0.1 mol·L^–1^ HNO_3_; II—40 μL of Pd solution was added to the three electrode-system; III—Pd was electrodeposited by chronoamperometry (potential applied: −0.7 V; interval time: 0.01s); IV—Pd nanoparticles are stabilized using 20 CV cycles. (C) Immunosensor assembly. I—Pd nanoparticles deposited on the surface of the working electrode; II—addition of CYS; III—Addition of GA; IV—addition of AntiSOD1 to generate selectivity against SOD1 target; V—addition of BSA to block free sites that did not interact with the antiSOD1; VI—addition of sample containing SOD1. Then, electrochemical measurements were performed to assess the concentration of the analyte.

Therefore, palladium electrodeposition (Figure 1B) occurred in two stages. The first, specifically for Pd electrodeposition, involved applying 40 μL of a 1.0 mmol L^–1^ Pd solution in 0.1 mol·L^–1^ HNO_3_ to the sensor surface, covering all three electrodes. Chronoamperometry was used to determine the optimal electrodeposition time, which will be discussed in Section 3.1. The chosen potential was −0.7 V, based on the work of Orzari et al.,^19^ which demonstrated a satisfactory potential for Pd electrodeposition on carbonaceous surfaces. In the second stage, using only 40 μL of 0.1 mol·L^–1^ HNO_3_, the Pd nanoparticles were stabilized by CV in a potential window of −0.5 to 0.9 V, v = 50 mV.s^–1^, for 20 scans.

Immunosensors can be assembled using different immobilization techniques, effectively forming a bond between the electrode surface and the desired antibody or antigen.^28,29^ In this case, the bond between CYS and GA forms a rigid and stabilized network, linking the antibody or antigen to the metals present on the electrode surface.^30^ In this work, this bond is formed through the use of Pd nanoparticles. Figure 1C shows the proposed mechanism of the immunosensor assembly. Video S2 shows the step-by-step process of the electrodeposition of Pd and the immobilization of biological materials.

### 2.5 Sample Preparation

5–HT determination was performed in commercial human serum (Sigma-Aldrich), using the addition and recovery method. The serum was diluted in 0.1 mol·L^–1^ PBS, pH 7.5, in a 1:100 (v/v) proportion. The 5–HT concentrations were achieved from a 10 mmol L^–1^ 5–HT stock solution (the data is present in the Supporting Information). The same human serum/PBS was also used to construct the analytical curve for SOD1. The dilution of biological samples such as, e.g., human serum or other animals, in detection using biosensors, given the complexity of the samples that can affect analytical measurements, as well as to minimize nonspecific binding.^31^

## 3 Results and Discussion

### 3.1 ePAD Characterization

ePADs can be fabricated using a wide range of paper substrates. Among the various options available and their distinct properties, textured office paper was selected for this study. Notably, the fiber agglomerates within the sheet contribute to its natural impermeability. However, during initial tests, we observed some leakage after longer use periods. To create a substrate capable of maintaining impermeability over several hours, which is essential for reliable electrochemical measurements and the reproducible immobilization of biological materials, we applied a beeswax coating to waterproof the paper. Oliveira et al.^11^ developed a dispersion of beeswax in hexane for conductive inks and concluded that it dispersed satisfactorily, forming a uniform film.

As outlined in Section 2.3, the beeswax waterproofing treatment substantially enhanced the substrate’s resistance to fluid permeation, allowing for extended retention of aqueous samples on the sensor surface without observable leakage. Notably, this modification did not introduce significant changes to the electrochemical response. It is important to note that the beeswax modification facilitates the final disposal of the sensor after use. When designing a system capable of disposal after electrochemical measurements, components that ensure the lowest possible environmental impact are required. In line with this idea, the beeswax coating has proven to be excellent as a waterproofing agent for the paper substrate, as it is a natural product that causes no damage after disposal and avoids the generation of environmentally harmful waste. While it is difficult to achieve 100% environmentally friendly disposal, it is important that new efforts are made to develop devices that cause as little damage as possible after measurement. Regarding the stability of the coating, the sensors produced can be used for a long time after manufacture, without the beeswax losing its waterproof properties, just as the ink does not lose its electrochemical performance. However, given the simplicity of the system, reusing the sensors is not possible, requiring disposal after electrochemical measurement. Figure 2A,B present SEM images of the paper substrate before and after beeswax modification. In the unmodified sample (Figure 2A,A′), a network of interwoven fibers typical of textured office paper is observed, with visible interfiber spaces facilitating liquid absorption. Furthermore, the intrinsic composition of this paper allows for short-term retention of the solution without immediate leakage, making it a viable platform for initial sensor applications before waterproofing.^8^

**Figure 2:**
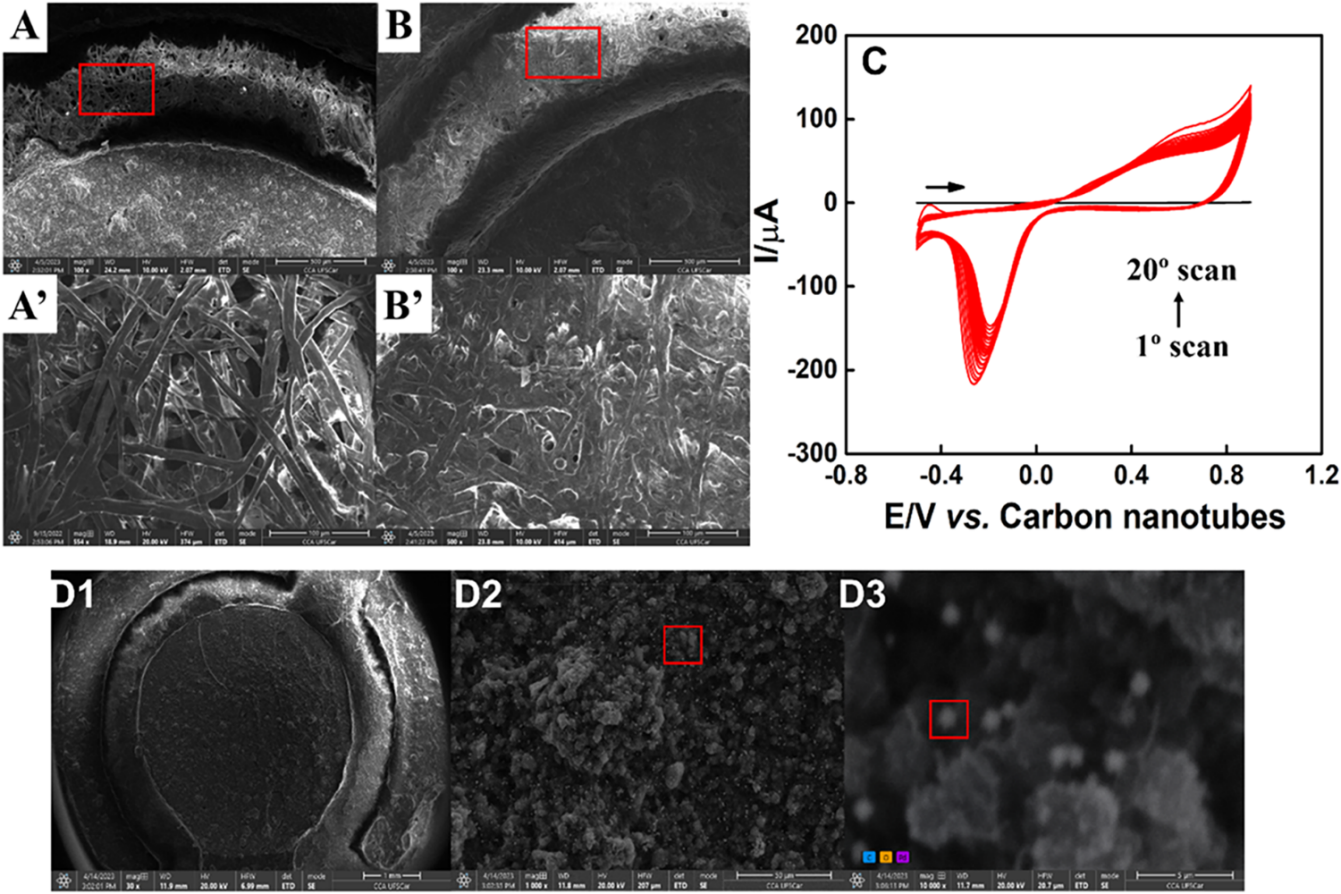
SEM images of the paper surface (A) without and (B) with beeswax at a magnification of 100×. A′ and B′ display a magnification of 500×. (C) CV (electrodeposited for 5 min) about this Pd stabilized on the Pd-CNTs-GV/ePAD surface in the presence of 0.1 mol·L^–1^ HNO_3_, v = 50 mV.s^–1^. (D) SEM images with magnification of (D1) 30, (D2) 1000 and (D3) 10,000×.

Following beeswax modification (Figure 2B,B′), the paper fibers are fully coated, resulting in a smoother surface and a significant reduction in porosity between fibers. The formation of a thin beeswax layer provided hydrophobic properties to the substrate, effectively preventing the infiltration of aqueous solutions. This surface modification was essential for maintaining solution stability during electrochemical measurements.

The working electrode’s surface was modified with Pd to enhance its analytical performance. In developing electrochemical immunosensors, incorporating metallic materials onto the working electrode surface is a widely adopted strategy, with gold and gold nanoparticles being the most commonly employed. This preference largely stems from the favorable interaction between gold and commonly used cross-linkers (e.g., CYS and GA), which can promote efficient binding of biomolecules.^30,32^

The optimization of electrodeposition time for Pd was examined between 1 and 10 min using chronoamperometry, followed by stabilizing the particles through CV. This procedure aimed to ensure the deposition and stabilization of Pd particles, preventing external factors from altering their behavior after prolonged environmental exposure.^33^ Stabilization studies are typically conducted using consecutive cycles to observe the metal’s behavior on the surface. The reduction in peak current magnitudes after successive cycles may relate to the detachment of smaller particles and the deposition of larger ones. In this context, stabilization was performed according to the work of Orzari et al.^33^ and Kaptoge et al.^34^ To decrease production time, stabilization was conducted over 20 cycles. Figure 2C presents the voltammogram for Pd stabilization after 20 cycles, which is deemed the ideal parameter. Although the stabilization between scans shows a greater difference in the initial cycles, by the 20th scan, there is a less pronounced difference between them. The value of 20 scans was also chosen to standardize the number of cycles.

SEM images and EDS data were acquired to determine the optimal Pd electrodeposition time. These images allowed for the visualization of Pd particles on the electrode surface and provided a semiquantitative analysis of the atomic percentage of Pd. Figure 2D presents SEM images of the CNTs–GV/ePAD at magnifications of 30×, 1000×, and 10,000×. At 1000× magnification (Figure 2D-2), small whitish dots indicative of Pd particles are visible. At 10,000× magnification (Figure 2D-3), spherical palladium units are attached to the carbon-based paint material. Detailed EDS spectra for each electrodeposition time (Figures S3 for 1 min to S9 for 10 min) and their corresponding atomic percentages are provided in the Supporting Information. Figure S10 presents a dot plot illustrating the relationship between electrodeposition time and %Pd atomic. Five minutes was chosen as the ideal time because it presents the highest %Pd.

Subsequent electrochemical studies of the CNTs–GV/ePAD sensor, both before and after electrodeposition, were conducted using CV in a 1.0 mmol L^–1^ [Fe­(CN)_6_]^4–/3–^ solution in 0.1 mol·L^–1^ KCl. Figure 3A shows the electrochemical behavior before electrodeposition. An anodic peak at 0.334 V with a *I*_pa_ = 17.2 μA and a cathodic peak at −0.381 V with *I*_pa_ = 17.6 μA were observed, yielding a peak-to-peak separation (Δ*E*) of 715 mV and an *I*_pa_/*I*_pc_ ratio of 0.98.

**Figure 3:**
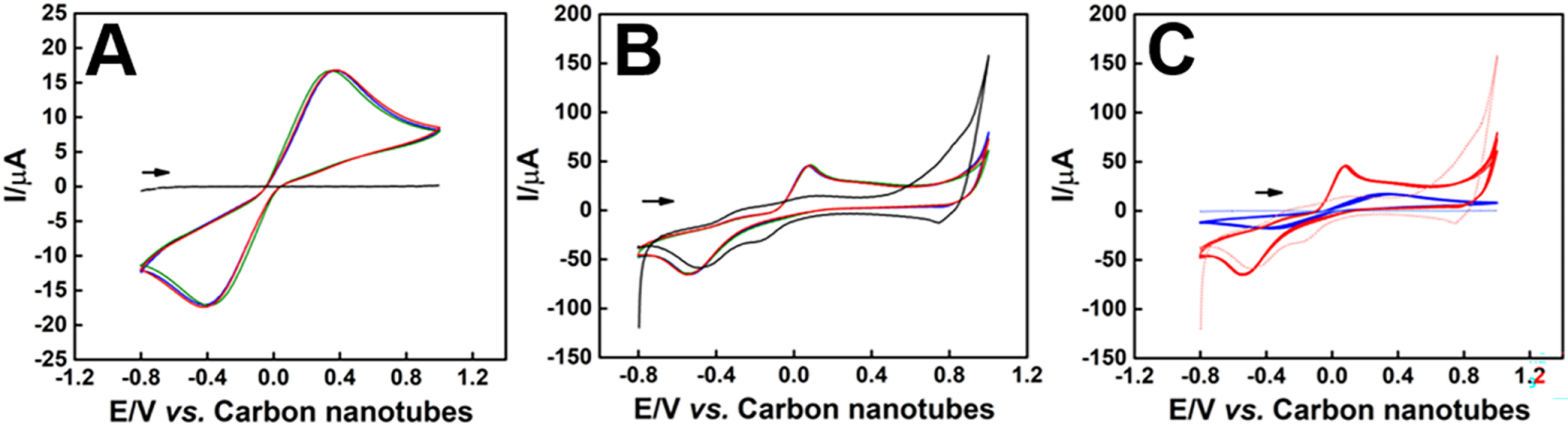
(A) CV of the CNTs-GV/ePAD in the absence of the Pd electrodeposited in the equimolar presence of 1.0 mmol L^–1^ [Fe­(CN)_6_]^4–/3–^ in 0.1 mol·L^–1^ KCl (red, blue, and green voltammogram) and in 0.1 mol·L^–1^ KCl (blank, black), v *=* 50 mV.s^–1^, *n* = 3. (B) CV of the Pd-CNTs-GV/ePAD in the equimolar presence of 1.0 mmol L^–1^ [Fe­(CN)_6_]^4–/3–^ in 0.1 mol·L^–1^ KCl (red, blue, and green voltammogram) and in 0.1 mol·L^–1^ KCl (blank, black), v *=* 50 mV.s^–1^, *n* = 3. (C) Comparison between the CV before (blue) and after (red) the Pd electrodeposited in the equimolar presence of 1.0 mmol L^–1^ [Fe­(CN)_6_]^4–/3–^ in 0.1 mol·L^–1^ KCl, v *=* 50 mV.s^–1^, *n* = 3.

Figure 3B presents the voltammograms after Pd electrodeposition. In the absence of the redox probe (black curve, 0.1 mol·L^–1^ KCl), no distinct anodic peak is observed, suggesting the formation of Pd oxides near 0.6 V. This oxide formation does not significantly influence the peak currents observed in subsequent voltammograms when the redox probe is present. When [Fe­(CN)_6_]^4–/3–^ is introduced, a notable anodic peak at 0.253 V with *I*_p_ = 34.0 μA appears, demonstrating a marked increase in electrochemical activity. This indicates that Pd electrodeposition significantly enhances the sensor’s response to the redox probe, which supports its use in future electrochemical characterizations without interference from Pd.

Regarding the cathodic peak, no significant difference in current is observed between the presence and absence of [Fe­(CN)_6_]^4–/3–^. This result, especially the prominent anodic peak in the presence of the redox probe and the minimal contribution of Pd to the overall current, confirms that [Fe­(CN)_6_]^4–/3–^ can be reliably used for electrochemical measurements in the immunosensor. This region is ideal for monitoring the electrochemical behavior after immobilizing biological materials, ensuring that the electrode material does not compromise the probe’s response.

Figure 3C compares the voltammograms before and after electrodeposition, highlighting the significant change in electrochemical behavior following the deposition of palladium.

### 3.2 Electrochemical Determination Of Serotonin

To investigate its electrochemical behavior, measurements were conducted using a 1.0 mmol L^–1^ 5–HT solution in phosphate-buffered saline (PBS, 0.1 mol·L^–1^, pH 7.5). For this first study using 5-HT, we sought to demonstrate the electrochemical behavior of the sensor after Pd electrodeposition, to observe analytical improvement, such as sensitivity in the presence of an analyte.

Figure 4A shows CVs comparing the electrochemical behaviors of sensors with and without Pd in the presence or absence of the analyte. Anodic peaks were obtained for both sensors, being positioned at 0.25 V and having a current of 49.17 μA for the Pd–CNTs–GV/ePAD sensor (CV, in red) and at 0.30 V, with a current of 16.07 μA for CNTs–GV/ePAD (CV, in blue). Therefore, the presence of Pd increased the anodic peak current more than 3 times, making clear the improvement of sensitivity in the presence of the metal. The behavior obtained for 5–HT is common for carbon-based sensors, with peaks near 0.3 V, in a reaction with 2 electrons and 2 protons (insert in Figure 4A), forming the quinone derivative.^35^

**Figure 4:**
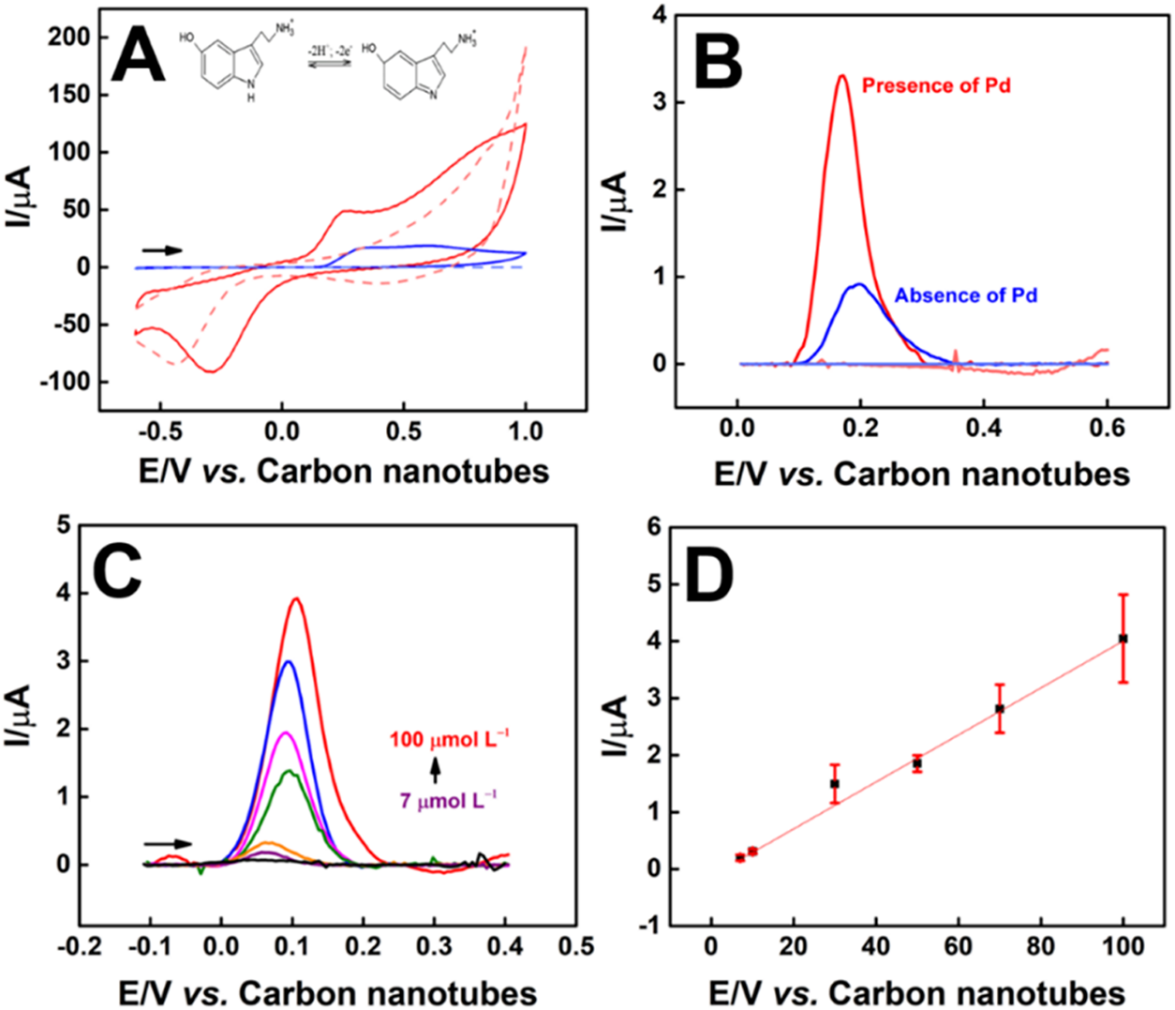
(A) Comparison between cyclic voltammograms of the CNTs-GV/ePAD (blue) and Pd-CNTs-GV/ePAD (red). The electrochemical measurements were performed in the presence of 1.0 mmol L^–1^ 5–HT in 0.1 mol·L^–1^ PBS (pH 7.5), v = 50 mV.s^–1^. The dashed voltammograms were performed 0.1 mol·L^–1^ PBS (pH 7.5) in the absence of analyte. The electrochemical oxidoreduction mechanism of the 5–HT is shown as the inset. (B) Comparison between the differential pulse voltammetry of the CNTs-GV/ePAD (blue) and Pd–CNTs–GV/ePAD (red). The electrochemical measurements were performed in 0.1 mmol L^–1^ 5–HT in 0.1 mol·L^–1^ PBS (pH 7.5). DPV parameters: amplitude = 100 mV, modulation time = 0.01 s, step = 5 mV, interval time = 0.017 s, v = 30 mV.s^–1^. (C) Differential pulse voltammetry of the Pd–CNTs–GV/ePAD obtained for different concentrations of 5–HT (7.00, 10.0, 30.0, 50.0, 70.0, and 100 μmol L^–1^). DPV parameters: amplitude = 100 mV, modulation time = 0.01 s, step = 5 mV, interval time = 0.017 s, v = 30 mV.s^–1^. (D) *I* vs *C*_5–HT_ correlation.

We also applied a pulsed technique to quantify 5–HT. Figure 4B shows differential pulse voltammograms for the sensor with and without Pd in 5–HT 0.1 mmol L^–1^ in 0.1 mol·L^–1^ PBS (pH 7.5). A significantly larger peak current is observed for the Pd–modified sensor, reaching 3.33 μA at 0.17 V, compared to 0.93 μA at 0.19 V for the unmodified sensor. Despite the slight shift in peak potential, the proximity of the peaks allows for a clear comparison, highlighting a more than 3-fold increase in signal magnitude and demonstrating the enhanced sensitivity of the Pd-modified sensor for 5–HT detection.

The analytical curve was constructed for 5–HT (7.00, 10.0, 30.0, 50.0, 70.0, and 100 μmol L^–1^) in 0.1 mol·L^–1^ PBS (pH 7.5). Figure 4C shows the voltammograms obtained for each of the concentrations. A linear correlation was obtained between the concentration of 5–HT and its peak current (Figure 4D), generating the equation *I*_p_(μA) = −1.20116 × 10^–7^ + 4.12613 × 10^–8^ × *C*_5–HT_ (μmol L^–1^), with an *R* ^2^ of 0.998. The limit of detection (LOD) was calculated using the following equation: LOD = 3.0·SD_blank_/slope, equal to 0.35 μmol L^–1^ in PBS (*n* = 9). Reproducibility was calculated using the relative standard deviation (RSD) with *n* = 6 in the presence of 5–HT 100 μmol L^–1^ in 0.1 mol·L^–1^ PBS, reaching a value of 8.03%.

As proof of concept for applying the Pd–CNTs–GV/ePAD sensor developed in this work, the recovery of 5–HT in a commercial human serum sample was carried out. A solution containing commercial human serum in 0.1 mol·L^–1^ PBS pH 7.5 (1:100 v/v) was spiked with known values of 5–HT equal to 100, 50, and 7 μmol L^–1^, with sample recoveries equal to 96.2, 102.3, and 108%, respectively, indicating that the sensor has the potential to determine the analyte in different concentrations, with recovery results close to the values added to the sample. Table S1 displays a comparison of electrochemical devices in the literature to determine 5–HT with the developed work. Bullapura Matt et al.^36^ developed a carbon paste electrode modified with zirconia oxide nanoparticles to determine 5-HT, with the device showing good reproducibility, high catalytic activity, and sensitivity for this application. Wu et al.^37^ modified a carbon-printed sensor using gold nanoparticles covalently linked to ferrocene in carbon nanotubes to improve the detection of 5-HT in urine samples in a sensitive, selective, and economical manner. Deepa et al.^38^ modified a carbon paste electrode with sodium citicoline to simultaneously determine dopamine and 5-HT. The modification favored separating the components’ peaks at 0.176 V, showing success in both determinations. Orzari et al.^39^ developed a disposable electrochemical sensor based on conductive automotive varnish paint and graphite to determine 5-HT. Silva et al.^40^ developed a new procedure involving sequential chemical treatment to generate reduced graphene oxide (rGO) inside 3D-printed poly­(lactic acid) (PLA) electrodes. They applied it in the determination of 5-HT. The printed device was used as a sensor and biosensor, showing good reproducibility, relatively low cost, and high sensitivity.

Table S2 shows the values added and recovered for each concentration. For the interference study (Figure S11), DPV measurements were performed for the presence of some species concomitant with the presence of 100 μmol L^–1^ of 5-HT. To construct the analytical curve, 5-HT was diluted in 0.1 mol·L^–1^ PB in the presence of 0.1 mol·L^–1^ KCL and 0.1 mol·L^–1^ NaCl, to ensure the salinity of the buffer. For the interferent study, Pd-CNTs-GV/ePAD was tested for three different media to observe its behavior when applied for the determination of 5-HT in the presence of saturated salts and glucose. Thus, a high concentration of KCl and NaCl salts were tested, being, at first, 0.1 mol·L^–1^ PB in the presence of 0.1 mol·L^–1^ KCl and 1.0 mol·L^–1^ NaCl, that is, a 10× increase in the presence of Na and, subsequently, 0.1 mol·L^–1^ of PB in the presence of 1.0 mol·L^–1^ KCl and 0.1 mol·L^–1^ NaCl (10× increase in K). As a last interferent, 100 μmol L^–1^ glucose in 0.1 mol·L^–1^ PBS (0.1 mol·L^–1^ of KCl and NaCl) was used. It is possible to observe that the presence of glucose and saturated salts alter the current magnitude in the determination of 5-HT, reaching an interference that varies from 10 to 20% of the signal obtained in the construction of the analytical curve (Figure S11-A). Furthermore, a potential shift is observed in the presence of other compounds or saturates, with the voltammograms of the analytical curve showing the determination of 5-HT appearing close to 0.10 V. For the interferents, the potential shifts to 0.17; 0.21; 0.24 V for glucose, sodium saturation, and potassium saturation, respectively (Figure S11–B). These potential values are also related to serotonin determination work as mentioned previously.

In this context, a wide range of electrochemical sensors applied in determining 5-HT can be noted. Thus, as a potential alternative for this application, the Pd–CNTs–GV/ePAD sensor appears as an easily obtainable device. Considering the premise of using Pd as an alternative to gold-modified sensors, it is worth highlighting its characteristics that make it attractive for use in sensors and biosensors. Among the characteristics that make sensors modified with gold nanoparticles unique are a high surface area-to-volume ratio, magnetic, plasmonic, and fluorescent properties, easy and rapid synthesis, and, when used in a controlled manner, lower toxicity than other nanoparticles.^41^ Compared to the properties of noble metal nanoparticles, palladium exhibits characteristics and properties similar to other metals, but stands out primarily for its extremely interesting physical and chemical characteristics, such as thermal stability, photocatalytic activity, high chemical stability, and electrical and optical characteristics.^42^ Thus, noble metal nanoparticles can be chosen according to application interest and clinical demand. For sensors, these materials amplify their potential when used as modifiers. Here, Pd nanoparticles also appear to have a relatively low cost and higher oxidation activity compared to gold and platinum.^43^ In this sense, regarding the application performed in this work, using Pd as a modifier for 5-HT detection, the results obtained can be compared to recent studies using gold nanoparticles and other components for the same application. It is worth noting that the linear range values obtained in these studies and the LOD are comparable, even though the device presented in this work is simpler and requires fewer modifiers.

Wu et al.^37^ used gold nanoparticles, ferrocene, and carbon nanotubes to modify a commercial screen-printed sensor for 5-HT detection, presenting a linear range of 0.05–20 μmol L^–1^ and LOD of 0.017 μmol L^–1^. Misia et al.^44^ detected 5-HT in plasma using a molecularly imprinted polymer layer, gold nanoparticles, and carbon nanotubes, presenting a linear range of 2.5–20 μmol L^–1^ and LOD of 1.7 μmol L^–1^. Ustundag et al.^45^ synthesized 4-aminothiophenol-linked gold nanorods in carbonized coal tar pitch, presenting a linear range of 0.5–10 μmol L^–1^ and LOD of 0.03 μmol L^–1^. Li et al.^46^ developed a label-free electrochemical gold aptasensor for 5-HT detection based on the specific binding between the 57-base aptamer and the analyte; the sensor showed a linear range of 1–100 μmol L^–1^ and LOD of 0.3 μmol L^–1^. Thus, it is clear that this work presented comparable linear range and LOD, but that these could be improved in the future to increase sensitivity. However, it presents itself as a simple and stable alternative to sensor modifications, as well as an alternative to systems that use gold nanoparticles with the addition of other components or relatively more complex production methodologies.

Although it uses CNTs, which can have a relatively high cost, it still has a total value per sensor unit of approximately 0.076 dollars. The values of the materials used are described in Table S3, converted from the Brazilian Real to the dollar, following the monetary quotation of the Central Bank of Brazil on April 23, 2025 (1.00 Brazilian Real = 0.1758 dollar). In this sense, the sensor demonstrates analytical results comparable to other works in the literature and is positioned as a relatively cheap alternative for this purpose

### 3.3 Electrochemical Immunosensor for SOD1 Detection

To observe the behavior of each immunosensor immobilization step, tests were performed separately for each interaction until the binding of interest between the surface with the immobilized materials and the analyte SOD1 was obtained. Thus, the spectra for the ePAD with Pd, Pd-CYS, Pd-CYS-GA, Pd-CYS-GA-AntiSOD1, Pd-CYS-GA-AntiSOD1-BSA and Pd-CYS-GA-AntiSOD1-BSA-SOD1 were obtained separately. Between each step, 0.1 mol·L^–1^ PBS, pH 7.5 was used to clean and remove excess modifiers. Procedures present in the literature were used as the basis for the established protocol.^30,47^ The redox probe 1.0 mmol L^–1^ [Fe­(CN)_6_]^4–/3–^ in 0.1 mol·L^–1^ KCl was used for EIS measurements. The anodic half-wave potential of the electrochemical probe was used to perform the analyses, equal to 0.0 V.

For each stage, the increase in charge transfer resistance (*R*_ct_) occurred due to the formation of monolayers of the added compounds. Along with the increase in the amount of material on the electrode surface, interactions between them also occur, leading to a blockage of the current flow stage. Figure 5A shows the EIS spectra for each immobilization step (*n* = 3). An increase in the diameter of the semicircle is observed, indicating an increase in *R*_ct_. An insert in Figure 5A illustrates the Randles circuit and the Warburg impedance, which aligns well with the data from the Nyquist diagrams. Figure 5B presents a dot plot showing each immobilization step’s *R*_ct_ averages and standard deviations (*n* = 3). The anticipated behavior of the device in detecting SOD1 was achieved, demonstrating recognition between the biological materials.

**Figure 5:**
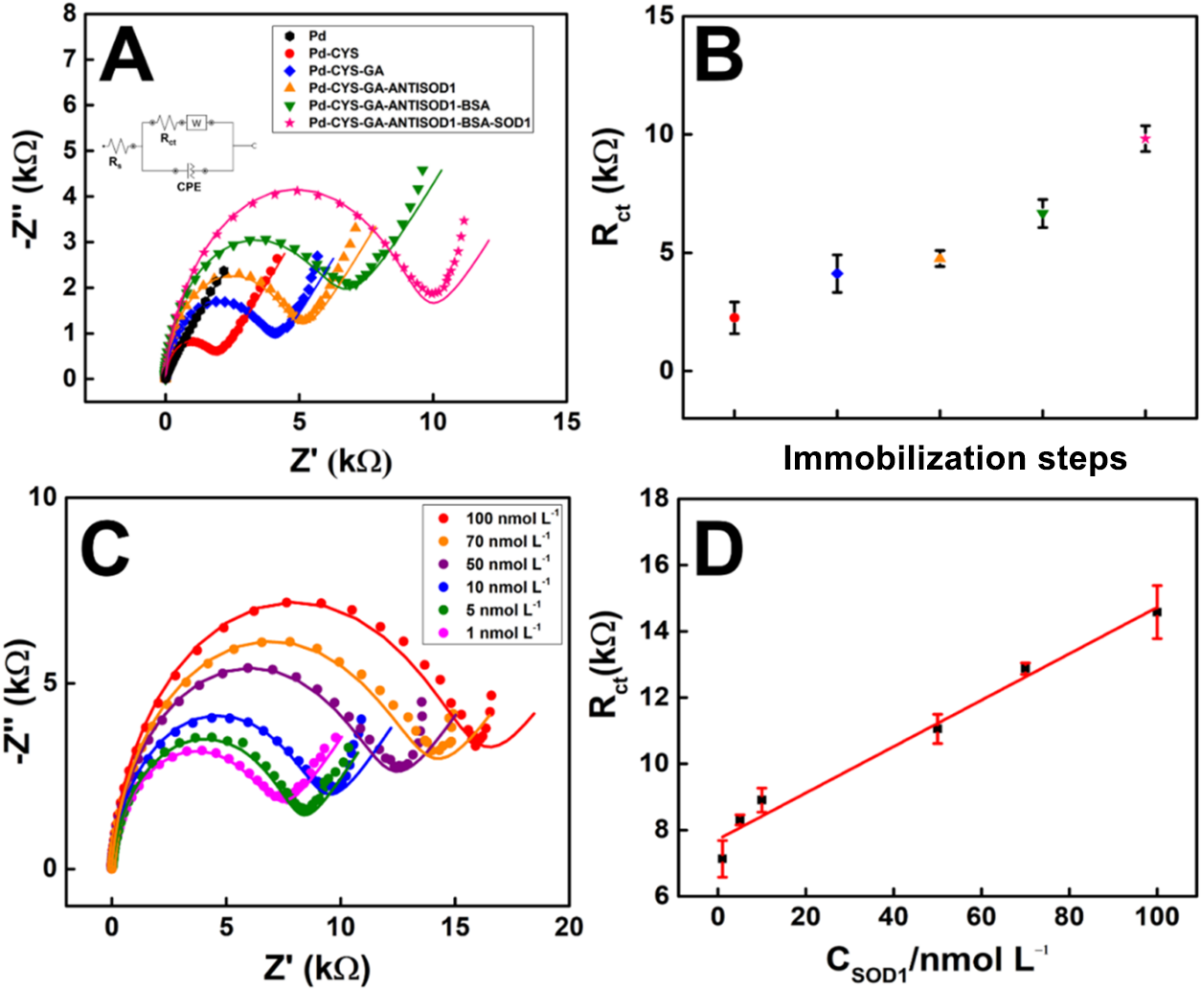
(A) Nyquist diagrams obtained by different modifications of Pd-CNTs-GV/ePAD, in the presence of [Fe­(CN)_6_]^4–/3–^ in 0.1 mol·L^–1^ KCl; The diagrams were obtained utilizing the *E*_1/2_ = 0 V; data presented are normalized on both axes. *Insert* of the Randles circuit (B) Graphic dot of each step immobilization step, being: CYS (red), CYS–GA (blue), CYS–GA–AntiSOD1 (orange), CYS–GA–AntiSOD1–BSA (green), and CYS–GA–AntiSOD1–BSA–SOD1 (pink). (C) Nyquist diagrams obtained by the modified SOD1–BSA–AntiSOD1–GA–CYS–Pd–CNTs–GV/ePAD with different concentrations of SOD1 in diluted human serum (1:100 v/v in 0.1 mol·L^–1^ PBS, pH = 7.5) (1.00; 5,00; 10.0; 50.0; 70.0 and 100 nmol L^–1^), in the presence of 1.0 mmol L^–1^ [Fe­(CN)_6_]^4–/3–^ in 0.1 mol·L^–1^ KCl, *E* = 0.0 V. Data presented are normalized on both axes. (D) *R*_ct_ vs *C*_SOD1_ correlation.

Studies were conducted to determine the optimal conditions for the immobilization of anti-SOD1 and its interaction with SOD1. The first test performed was the optimization of the anti-SOD1 concentration. The concentration of the bioreceptor ranged from 0.01 to 1.00 mg.mL^–1^, with a 30 min incubation time. Figure S12-A displays the EIS spectrum for the different antibody concentrations. Figure S12–B presents a dot plot relating the *R*_ct_ values obtained for each concentration from their respective Nyquist plots, indicating the means and standard deviations of each antiSOD1 concentration after the addition of SOD1. The *R*_ct_ values obtained were 5.7 ± 1.6, 4.7 ± 0.1, and 4.2 ± 1.0 kΩ for 0.01, 0.1, and 1.0 mg.mL^–1^ of antibody, respectively. It is noteworthy that, for EIS, the concentration of 1.00 mg.mL^–1^ yielded the highest Rct, making it the ideal choice for further studies.

The study of the anti-SOD1 immobilization time varied from 30 to 90 min (*n* = 3). All *R*_ct_ was obtained after the addition of the SOD1 sample. The mean and standard deviation values for *R*_ct_ obtained were 7.8 ± 1.5, 12.3 ± 3.5, and 8.0 ± 3.3 kΩ, respectively. Figure S12-C shows the EIS spectra for each time studied, while Figure S12-D shows the mean *R*_ct_ values. In this sense, it is possible to note a higher *R*_ct_ for the 60 min immobilization. Although the standard deviation of the mean for this time is relatively high, its value is expressively higher than for the other tested times. Therefore, the ideal condition for assembling the immunosensor was 1.0 mg.mL^–1^ antiSOD1 and an immobilization time of 60 min.

An analytical curve was constructed in 0.1 mol·L^–1^ PBS, pH 7.5, showing a linear relationship between Rct and SOD1 concentrations ranging from 1.0 to 100 nmol L^–1^ (Figure S13-A). The linear regression produced the equation *R*_ct_ (kΩ) = 22.9 (±0.942) + 1.8 (±0.12) *C*_SOD1_ (nmol L^–1^), with an *R* ^2^ of 0.987 (Figure S13–B). The limit of detection (LOD) was calculated using the following equation: LOD = 3.0·SD_blank_/slope, as 0.72 nmol L^–1^ (*n* = 10).

To evaluate the potential of the developed biosensor for real application, an analytical curve was constructed in human blood serum diluted in 0.1 mol·L^–1^ PBS pH 7.5 (1:100). A linear relation between *R*_ct_ and SOD1 concentrations ranging from 1.00 to 100 nmol L^–1^. Figure 5C shows the EIS spectra obtained. Figure 5D shows the analytical curve and linear regression, with a straight-line equation equal to *R*_ct_ (kΩ) = 7.7 (±0.27) + 7 × 10^–10^ (±0.5 × 10^–10^) *C*_SOD1_ (nmol L^–1^), with an *R* ^2^ of 0.979.

For both curves, the linear range obtained comprises the concentration of SOD1 biological levels in humans, calculated from the molar mass of SOD1 of approximately 32 kDa.^48^ In a simple conversion, the LOD value obtained in this work would be approximately 23 ng·L^–1^. For healthy humans, SOD1 values in blood are distributed at 548 ng.mL^–1^ in serum, 173 ng.mL^–1^ in plasma, and 242 ng.mL^–1^ in erythrocytes, approximately.^48^ Thus, the results obtained for the determination of SOD1 can be considered satisfactory, given the relative simplicity of the system.

It is interesting to note that while there is a relationship between SOD 1 and neurodegenerative diseases, few studies have sought to determine this directly. Furthermore, they propose using more complex materials to create immunosensors for this application. Table 1 shows a comparison between the linear range and LOD of the device developed here and other relevant examples from the literature. Dated as one of the first label-free immunosensors for the determination of SOD 1, Santharaman et al.^49^ created an immunosensor utilizing a screen- printed carbon electrode modified with self–assembled monolayers of gold nanoparticles on electropolymerized polypyrrole. Dhinesh et al.^50^ modified a screen–printed carbon electrode by molecular imprinting with poly­(3–ammoniophenylboronic acid) to detect SOD 1. Thandavan et al.^51^ developed a nanointerfaced biosensor using a gold electrode modified with iron nanoparticles. Tang et al.^52^ created a sensor modified with platinum and palladium nanoparticles on chemically reduced graphene oxide coated with polydopamine, demonstrating a fast-response and simple system. Among these works, the Pd–CNTs–GV/ePAD sensor exhibits analytical performance comparable to that reported in the literature, including a linear range and LOD encompassing biologically relevant concentrations.

**Table 1:** Comparison between the Linear Range and LOD with Literature Works^a^

device	linear range (nmol L^–1^)	LOD (nmol L^–1^)	references
GNP/PPy/SPCE	0.005–5.00	0.005	^49^
SOD1MIPP3APBA/SPCE	1000–5 × 10^5^	400	^50^
SOD/NanoFe_3_O_4_/Au	0.200–1.40	0.003	^51^
SOD/PtPd-PDARGO	16.0–240	2.00	^52^
Pd-CNTs-GV/ePAD	**1.0–100**	**0.720**	**This work**

a GNP/PPy/SPCE: SPE electrode modified with self-assembled monolayers of gold nanoparticles in electropolymerized polypyrrole with monoclonal anti-SOD1; **SOD1MIPP3APBA/SPCE:** Screen Printed carbon electrode based molecularly imprinting sensors; **SOD/NanoFe**_**3**_ **O**_**4**_ **/Au:** superoxide dismutase immobilized on iron oxide nanoparticles coated on a gold electrode surface; **SOD/PtPd-PDARGO:** synthesis of PtPd nanoparticles on chemically reduced graphene oxide coated with polydopamine; **Pd-CNTs-GV/ePAD:** electrochemical sensor based in conductive ink with palladium electrodepositing on paper substrate.

In addition to the analytical results, it is interesting to compare the sensor’s behavior in the presence of SOD1 in PBS and, subsequently, in diluted human serum. As highlighted in the reason for the 100× dilution of human serum (Section 2.5), it is worth emphasizing that in the presence of serum, it was possible to better visualize the SOD1 concentrations in this matrix, which may correlate with a possible sample interference in the measurement. However, given the high complexity of human serum, as highlighted by the suppliers themselves, various components such as glucose, proteins, electrolytes, sodium, iron, hemoglobin, etc. are present in this type of sample.^26^ The presence of these components culminated in the construction of two analytical curves that highlight this interfering behavior and demonstrate the possibility of determination in both media.

Notably, the materials used in fabricating this sensor aim to provide a device with materials that are relatively easy to obtain and reproduce. In contrast to other studies that utilized commercial sensors and/or relatively expensive noble metals such as gold and platinum, the Pd–CNTs–GV/ePAD was entirely fabricated in the laboratory using conductive ink, a waterproofing solution, and an environmentally friendly substrate. This is particularly significant, as the resulting electrochemical sensor/immunosensor was constructed from accessible and cost-effective materials.

The study, which aims to potentially quantify SOD1 levels in human serum or other biological matrices, such as blood and cerebrospinal fluid, offers a tool for emerging palliative therapies for patients with neurodegenerative diseases. Typically, the neurodegenerative disease most associated with high levels of the SOD1 protein and its various mutations is amyotrophic lateral sclerosis.^53^ Since early measurement of SOD1 levels is the primary method for improving quality of life and life expectancy—allowing for rapid therapeutic action in the early stages—tools that directly determine these levels significantly influence therapies focused on reducing the protein’s levels.^54^ Moreover, this work supports an alternative method for biomarker detection, even if simple and inexpensive, to identify potential trends in high SOD1 levels, as it has a LOD that covers the range of healthy protein levels and a linear range aiding in accurate quantification.

The same contribution can be made to the simple serotonin detection developed in this work, with increased sensitivity using a simple Pd electrodeposition technique. Serotonin has both therapeutic and quantifiable functions, demonstrating the alert potential of a patient prone to developing neurodegenerative diseases. For both applications, the results obtained demonstrate linear ranges and LOD comparable to various devices in the literature, serving as an alternative due to its relatively low cost and ease of fabrication and application.

Additionally, it is worth noting that there are limitations to applications in real clinical samples, which require patient screening and more robust studies on real samples. In this work, we seek to demonstrate the real trend of applications in clinical samples, recovering analytical values in commercial human serum. Thus, we have a simple and rapid ePAD produced, with consistent results for the determination of biomarkers of neurodegenerative diseases, and which corroborates the search for the development of sensors and immunosensors with potential application in real clinical samples.

## 4 Conclusion

We developed a novel Pd-CNTs-GV/ePAD sensor for the detection of 5-HT and used it as the foundation for an immunosensor to detect SOD1 in diluted human serum samples. Utilizing textured office paper as a substrate, along with a low-cost, lab-fabricated conductive ink and beeswax-based waterproofing, showcases an accessible and environmentally friendly approach to sensor fabrication. The electrodeposition of palladium on the working electrode significantly improved the device’s electrochemical performance, offering a cost-effective and biocompatible alternative to gold-based materials. The sensor exhibited high sensitivity toward 5-HT and reliable analytical behavior for SOD1 detection, supported by reproducible calibration curves. Incorporating anti-SOD1 and a well-structured surface modification strategy further confirmed the immunosensor’s applicability for clinical biomarker analysis. The proposed platform provides a promising alternative to conventional diagnostic tools, combining affordability, ease of fabrication, reduced reagent consumption, and effective analytical performance. These attributes make it particularly appealing for point-of-care testing and applications in resource-limited settings, with potential for further development and integration into portable diagnostic systems.

## Supplementary Material







## Notes

Representative diagram of the beeswax sealing, a photograph of the electrode compared to a US coin and its dimensions relative to a ruler, a dot plot showing the atomic percentage of electrodeposited palladium, cyclic voltammetry characterization of serotonin, differential pulse voltammetry to measure serotonin in buffer and its corresponding analytical curve, an interferent study for serotonin, electrochemical impedance spectroscopy to study the immunosensor parameters for SOD1, an analytical curve for SOD1 in buffer, a table comparing the linear range and detection limit of the device with others in the literature, recovery values for serotonin in diluted human serum, a table of material costs to produce the ePAD, and EDS information to determine the atomic percentage of electrodeposited palladium (PDF)
lv_0_20251031114458.mp4 (Video S1) (MP4)
lv_0_20251031122600.mp4 (Video S2) (MP4)
