# Supplementary material for: Paper-Based Electrochemical Device Modified with Palladium: Sensor for the Detection of Serotonin and an Immunosensor for the Detection of SOD1
Source: ACS Omega. 2025 Nov 10;10(46):55887–97. doi: 10.1021/acsomega.5c07375 (PMC12658648; doi:10.1021/acsomega.5c07375)
Supplement: Supplementary file 1 [file ao5c07375_si_001.pdf]

Supplementary Information

Paper-based electrochemical device modified with palladium: sensor for the detection of serotonin and an immunosensor for the detection of SOD1

Jefferson H.S. Carvalho[a], Bruna S. Faria[a], Rafaela C. Freitas[a], Laís C. Brazaca[b], Bruno C. Janegitz[a]*

[a] *Federal University of São Carlos (UFSCar), Araras, 13604-900, SP, Brazil*

[b] *São Carlos Institute of Chemistry (IQSC), University of São Paulo (USP), São Carlos 13566-590, SP, Brazil*

Corresponding Author*: brunocj@ufscar.br

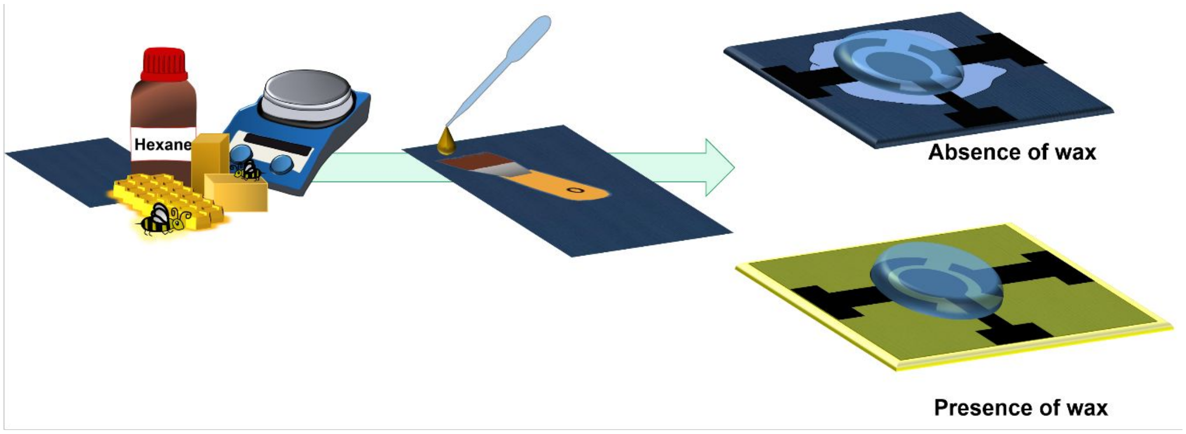

Figure S1. Schematic representation of the waterproofing of the paper by beeswax solution

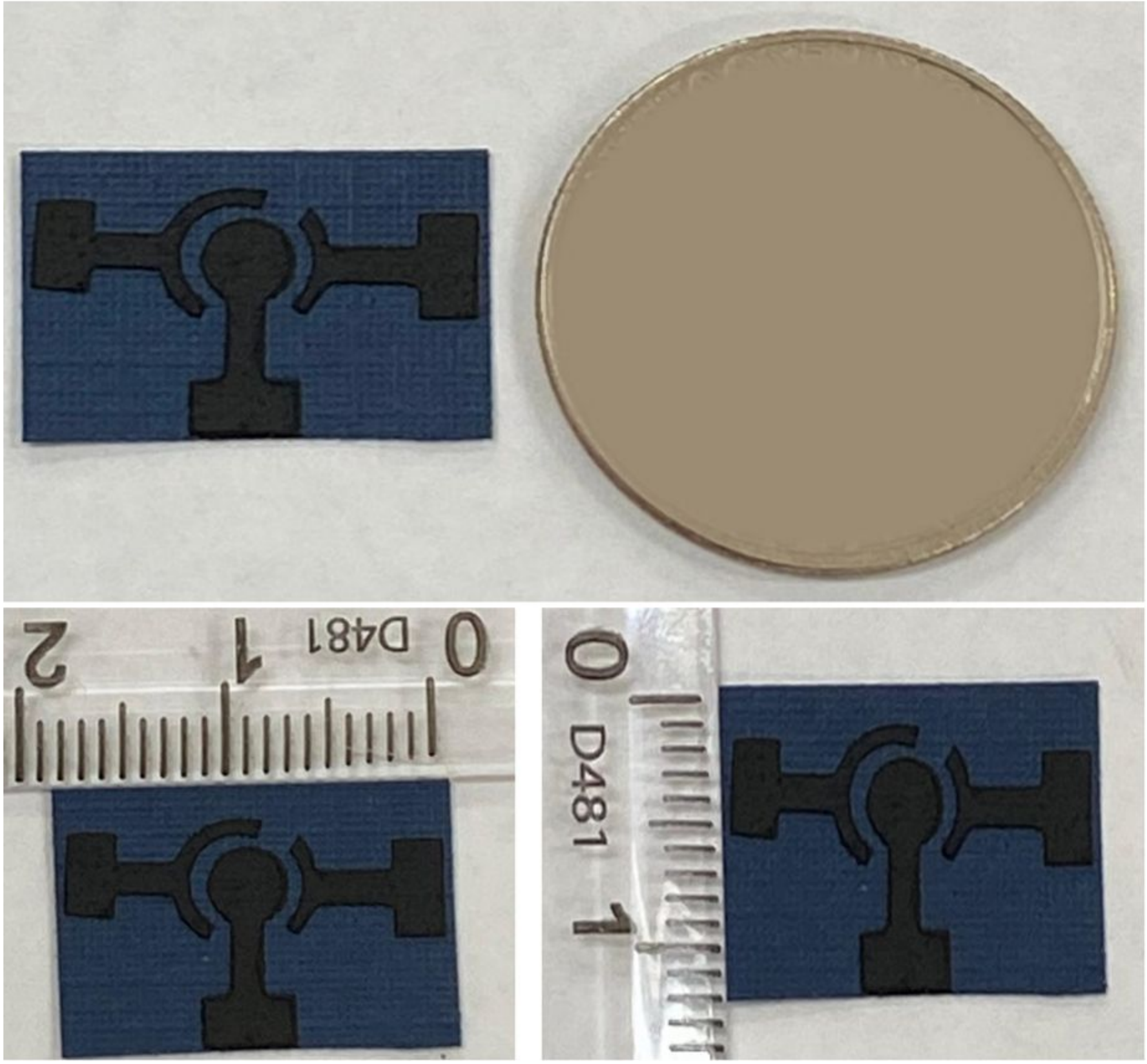

Figure S2. Comparison of the dimensions of the CNTs-GV/ePAD sensor with an American quarter dollar coin and a ruler

Figure S3 - EDS performed on the Pd-CNTs-GV/ePAD sensor after 1-minute electrodeposition of Pd solution of 1.0 mmol L^{-1} in HNO3 0.1 mol L^{-1} by chronoamperometry (potential applied: –0.7 V; interval time: 0.01s)

Session 23-04-14 14:12

Site 3

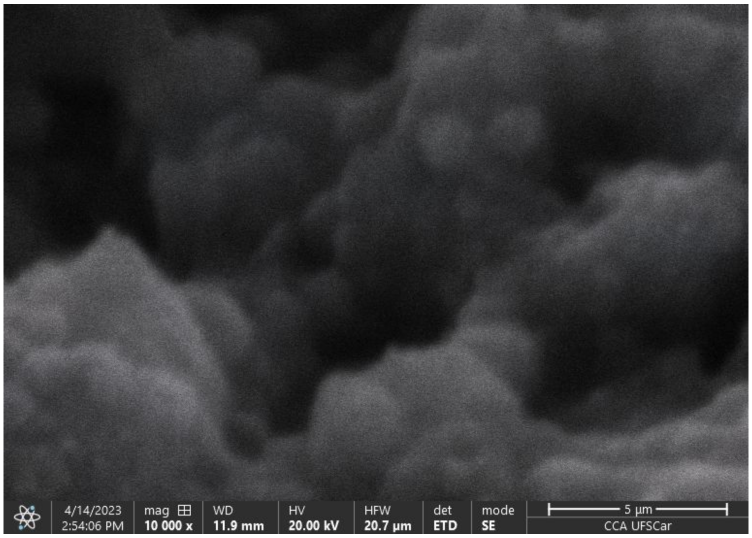

Map 1

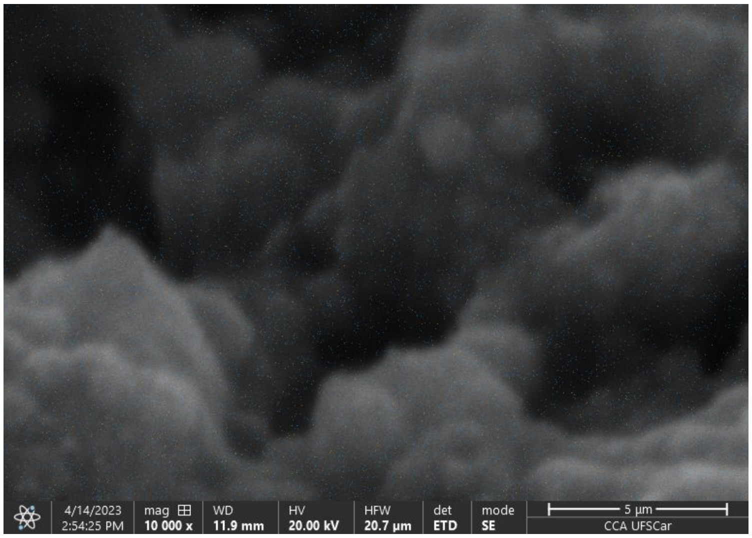

Total Number of Counts: 3001
Total Acquisition Time: 10 seconds
Average Count Rate: 300 cps
Acceleration Voltage: 20 kV
Map Resolution: 768 x 512

Element	Atomic %	Atomic % Error	Weight %	Weight % Error
C	77.2	0.7	71.8	0.6
O	22.8	1.4	28.2	1.7

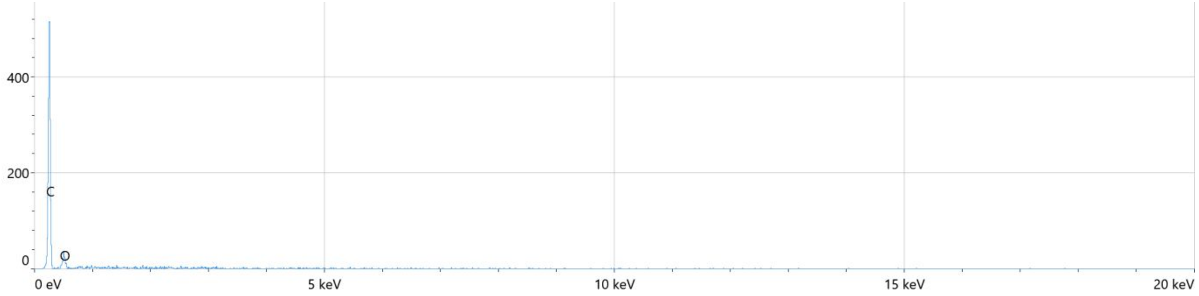

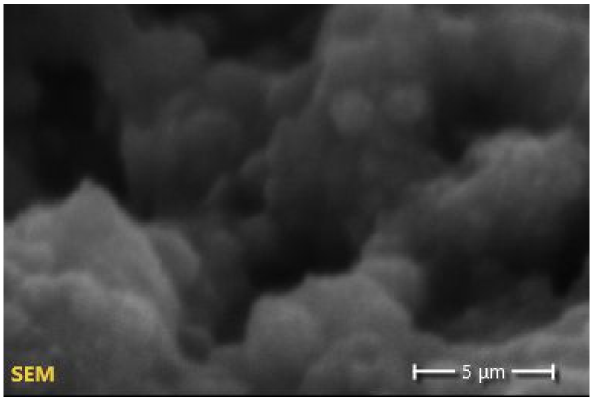

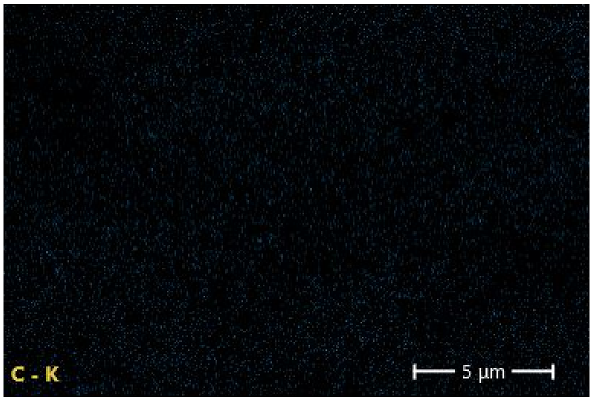

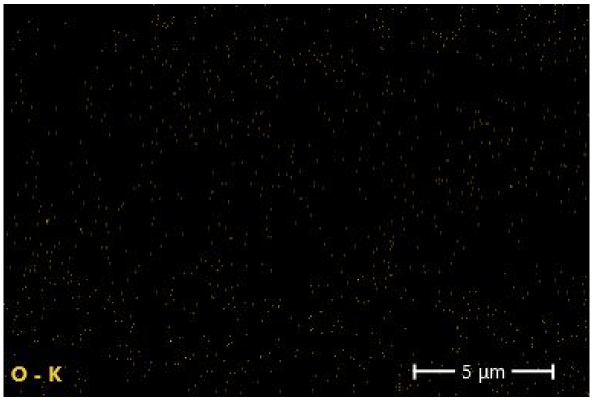

Figure S4- EDS performed on the Pd-CNTs-GV/ePAD sensor after 2-minute electrodeposition of Pd solution of 1.0 mmol L^{-1} in HNO3 0.1 mol L^{-1} by chronoamperometry (potential applied: –0.7 V; interval time: 0.01s)

Session 23-04-14 14:12

Site 1

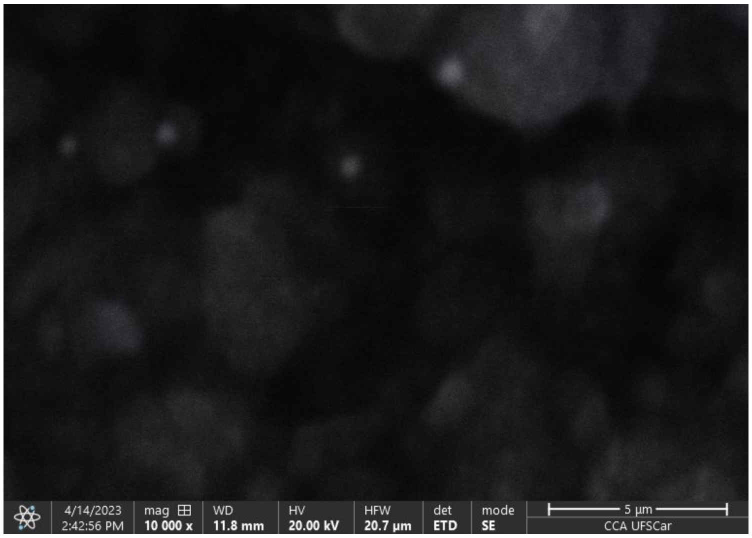

Map 1

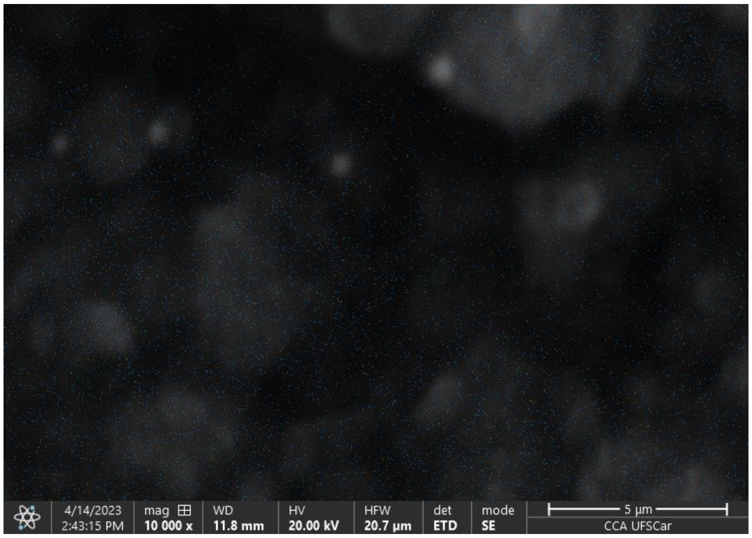

Total Number of Counts: 1912
Total Acquisition Time: 10 seconds
Average Count Rate: 191 cps
Acceleration Voltage: 20 kV
Map Resolution: 768 x 512

Element	Atomic %	Atomic % Error	Weight %	Weight % Error
C	89.0	1.1	83.4	1.0
O	10.6	1.5	13.2	1.9
Pd	0.4	0.1	3.4	0.7

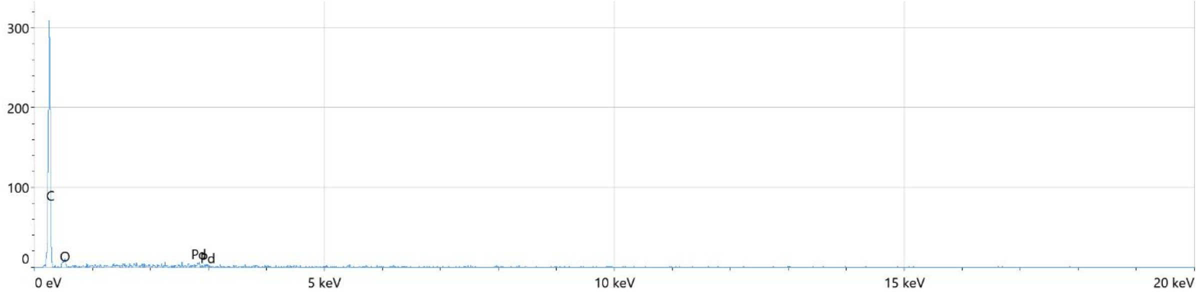

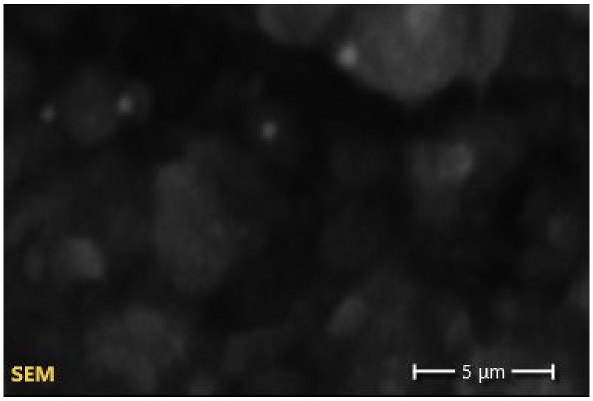

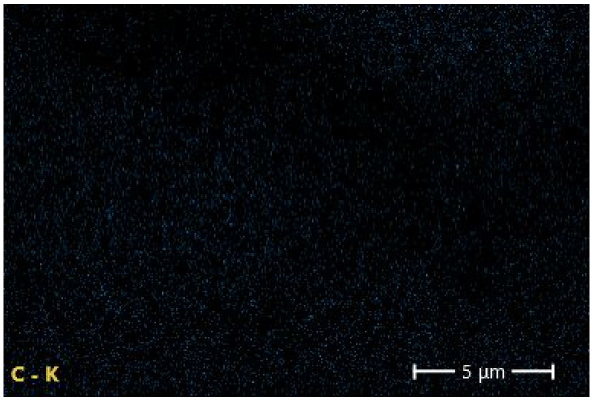

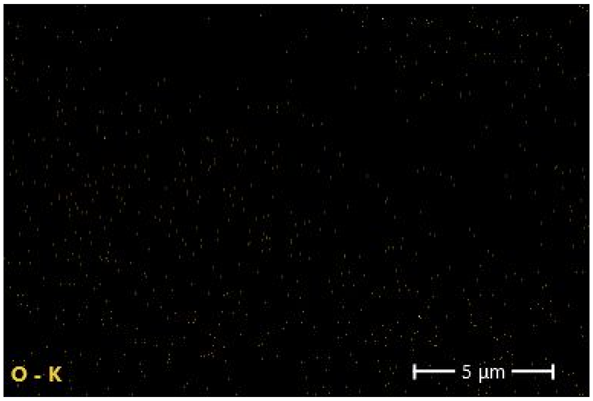
O - K
5 µm

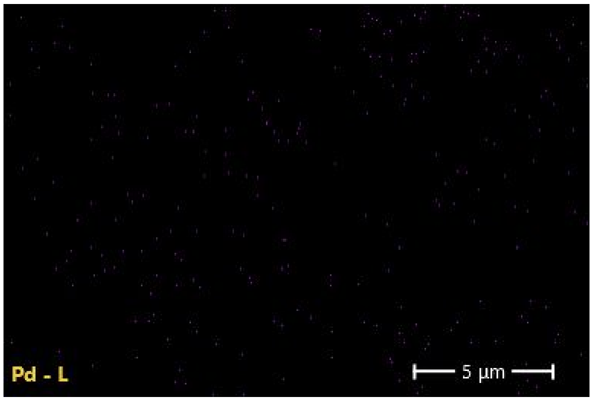
Pd - L
5 µm

Figure S5 - EDS performed on the Pd-CNTs-GV/ePAD sensor after 3-minutes electrodeposition of Pd solution of 1.0 mmol L^{-1} in HNO3 0.1 mol L^{-1} by chronoamperometry (potential applied: –0.7 V; interval time: 0.01s)

Session 23-04-14 14:12

Site 2

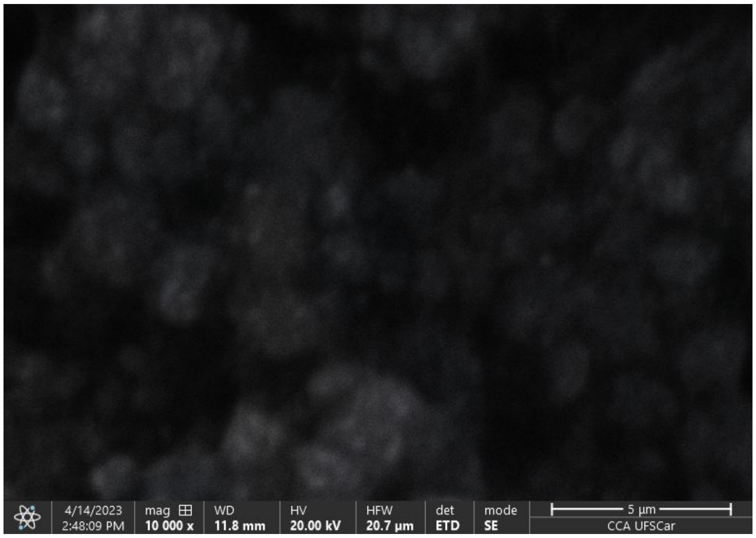

Map 2

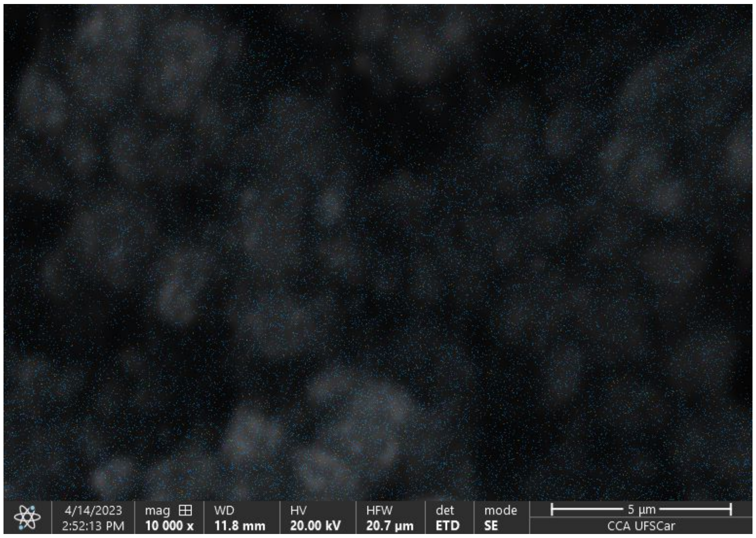

Total Number of Counts: 38040
Total Acquisition Time: 195 seconds
Average Count Rate: 195 cps
Acceleration Voltage: 20 kV
Map Resolution: 768 x 512

Element	Atomic %	Atomic % Error	Weight %	Weight % Error
C	80.8	1.1	72.1	1.0
O	18.4	0.5	21.9	0.6
Pd	0.8	0.0	6.0	0.3

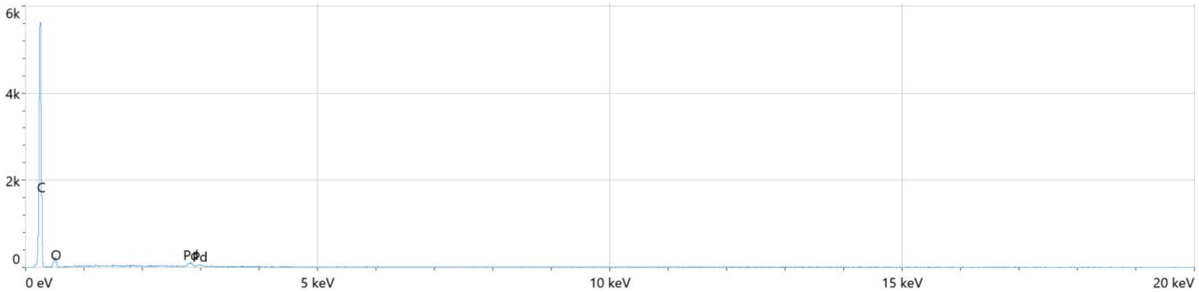

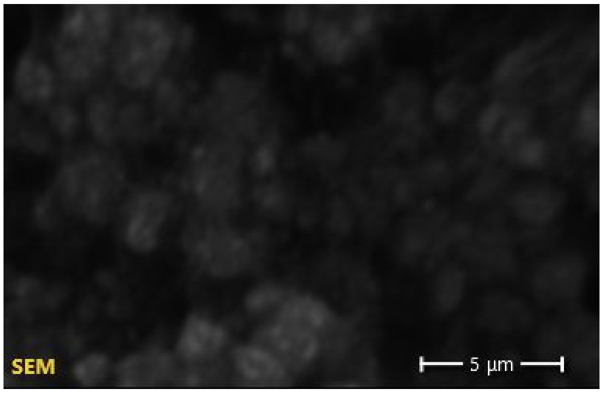

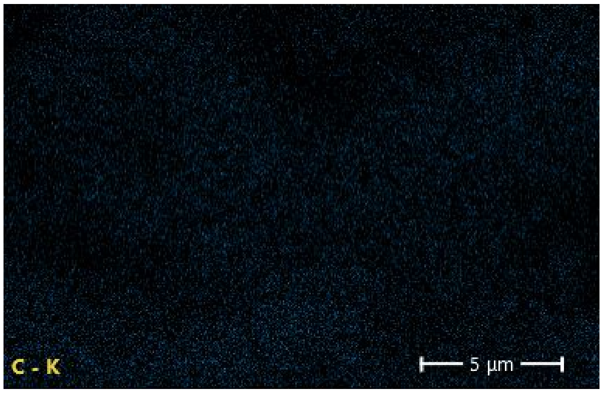

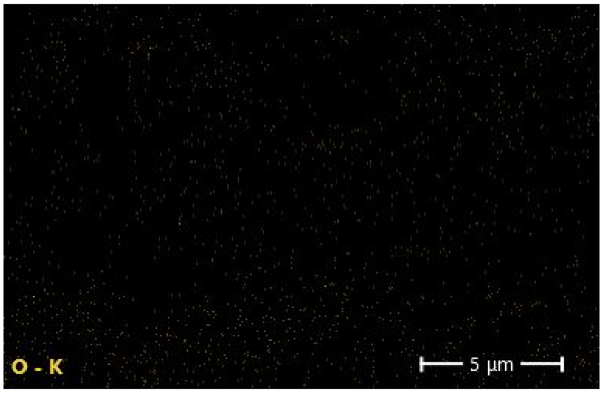

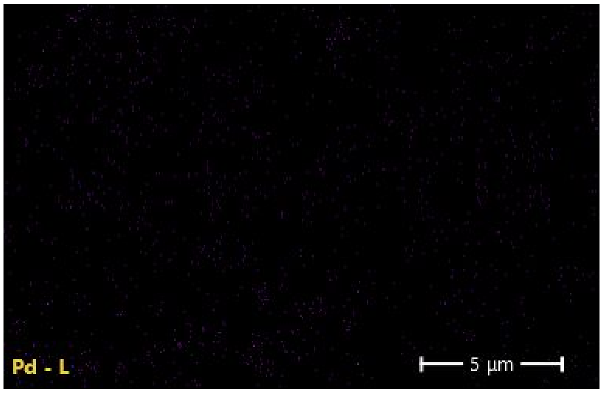
Pd - L
5 µm

Figure S6 - EDS performed on the Pd-CNTs-GV/ePAD sensor after 4-minutes electrodeposition of Pd solution of 1.0 mmol L^{-1} in HNO3 0.1 mol L^{-1} by chronoamperometry (potential applied: –0.7 V; interval time: 0.01s)

Session 23-04-14 14:12

Site 4

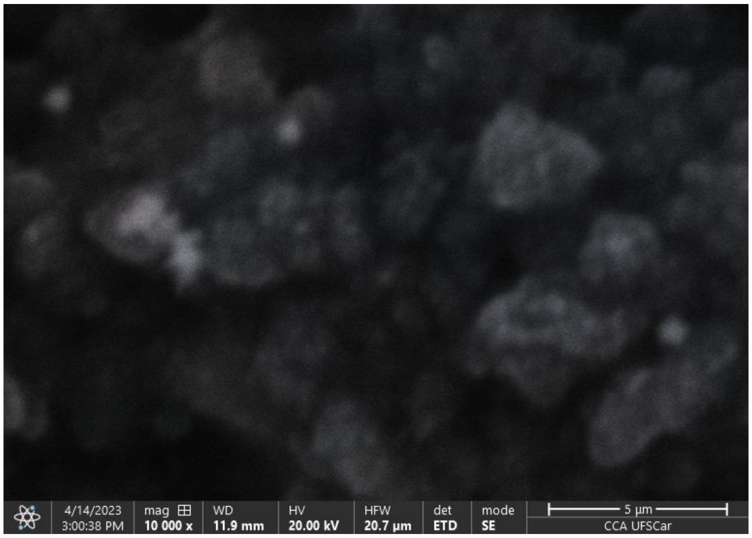

Map 1

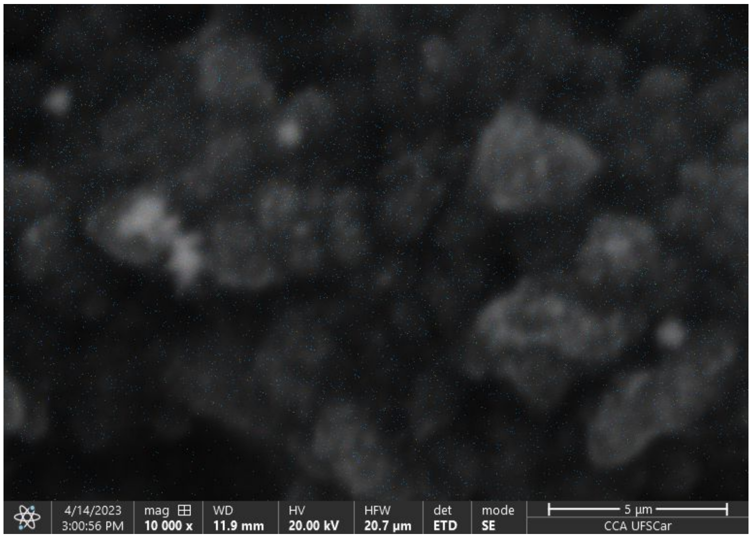

Total Number of Counts: 2083
Total Acquisition Time: 10 seconds
Average Count Rate: 208 cps
Acceleration Voltage: 20 kV

Map Resolution: 768 x 512

Element	Atomic %	Atomic % Error	Weight %	Weight % Error
C	80.9	0.9	71.1	0.8
O	18.1	1.6	21.2	1.9
Pd	1.0	0.1	7.7	0.4

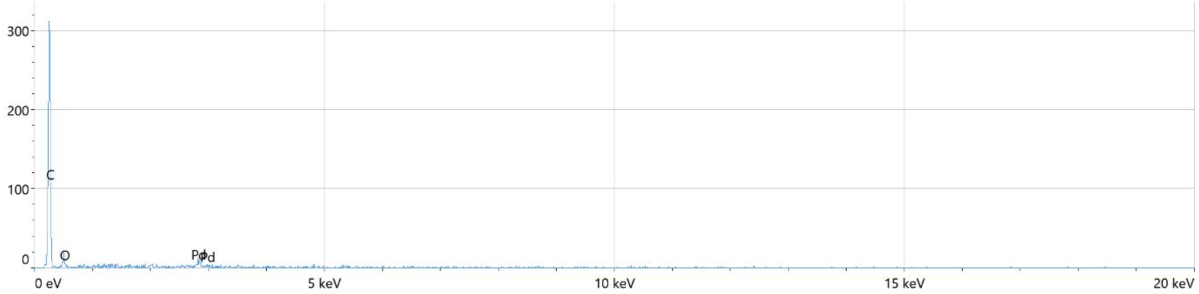

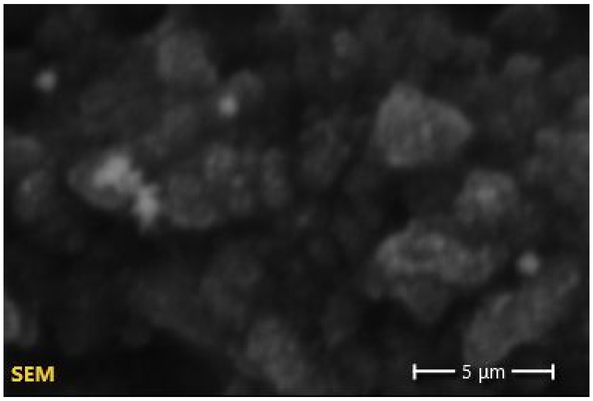

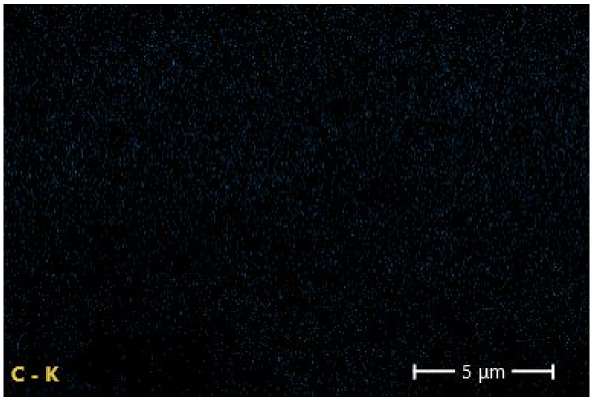

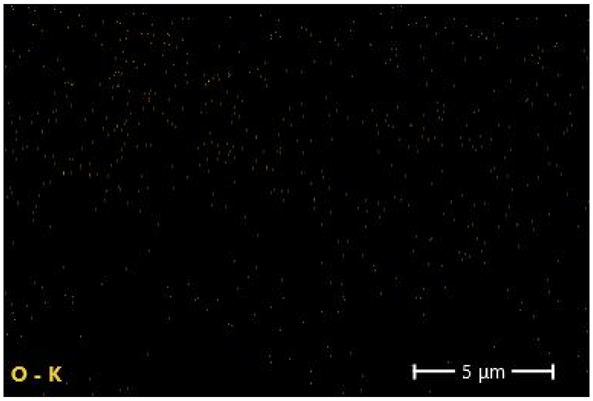
O - K
5 µm

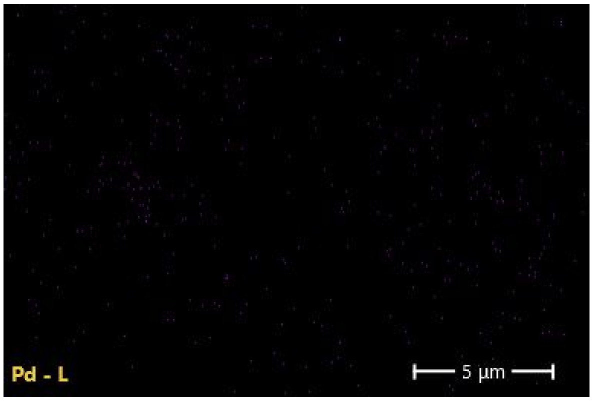
Pd - L
5 µm

Figure S7 - EDS performed on the Pd-CNTs-GV/ePAD sensor after 5-minute electrodeposition of Pd solution of 1.0 mmol L^{-1} in HNO3 0.1 mol L^{-1} by chronoamperometry (potential applied: −0.7 V; interval time: 0.01s)

Session 23-04-14 14:12

Site 5

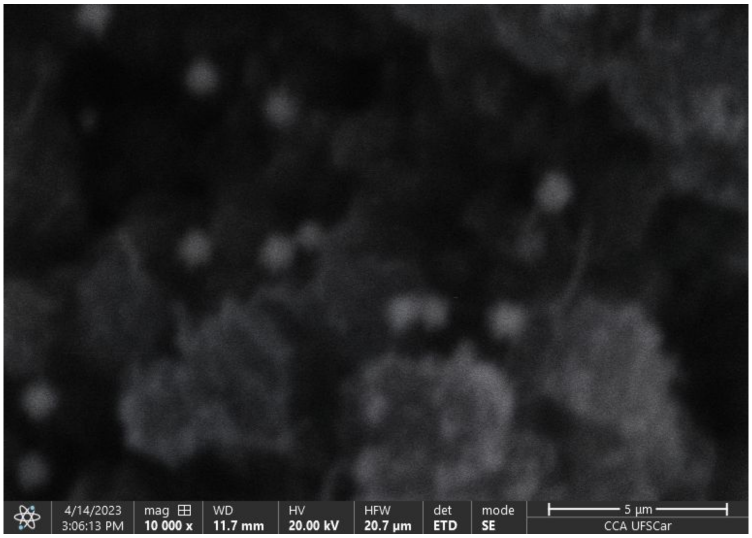

Map 1

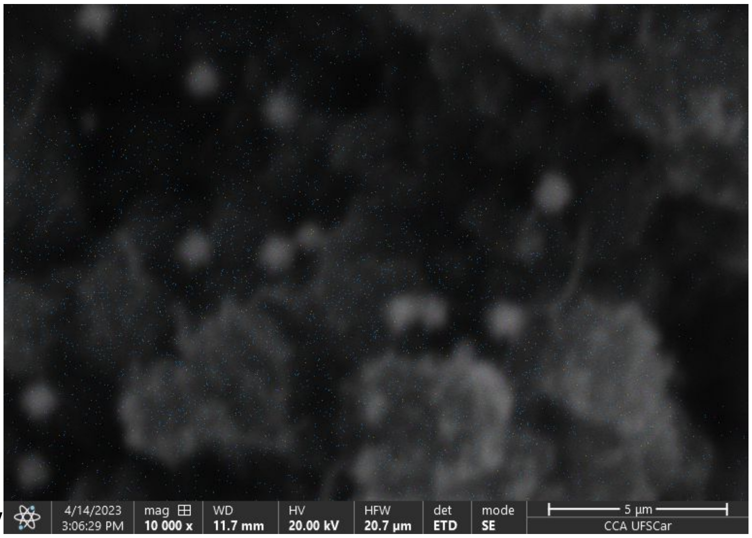

7

Total Number of Counts: 1874
Total Acquisition Time: 9 seconds
Average Count Rate: 208 cps
Acceleration Voltage: 20 kV
Map Resolution: 768 x 512

Element	Atomic %	Atomic % Error	Weight %	Weight % Error
C	81.4	1.1	67.1	0.9
O	16.6	1.5	18.2	1.7
Pd	2.0	0.1	14.6	0.5

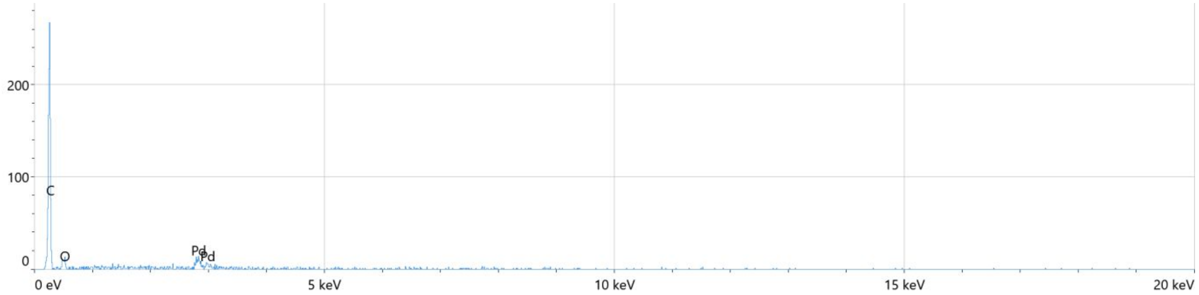

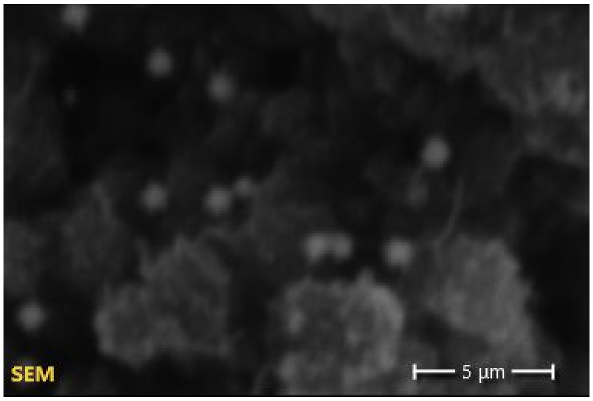

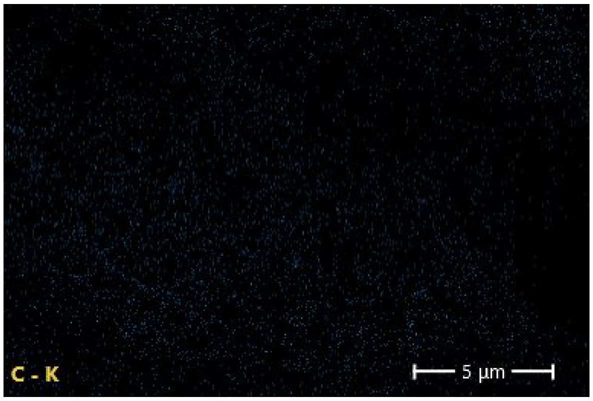

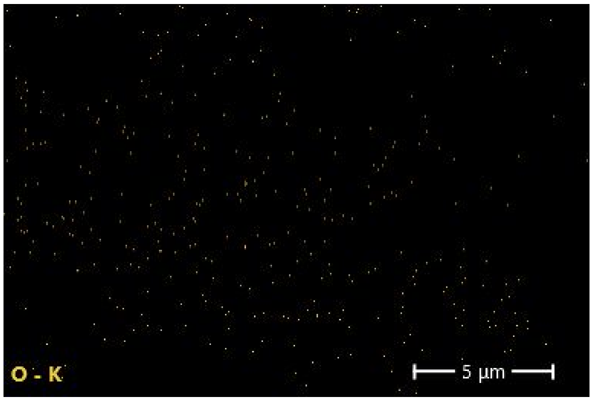
O - K
5 µm

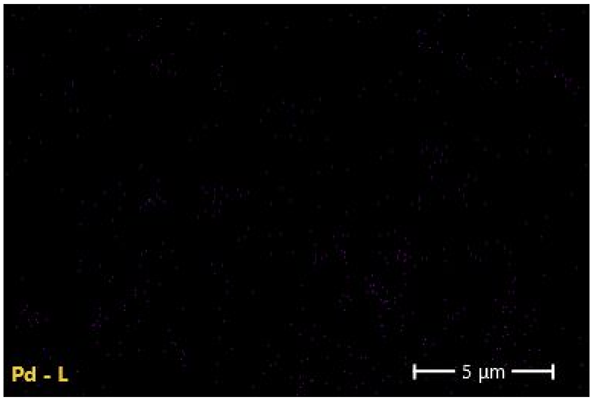
Pd - L
5 µm

Figure S8 - EDS performed on the Pd-CNTs-GV/ePAD sensor after 7.5-minutes electrodeposition of Pd solution of 1.0 mmol L^{-1} in HNO3 0.1 mol L^{-1} by chronoamperometry (potential applied: –0.7 V; interval time: 0.01s)

Session 23-05-04 14:20

Site 1

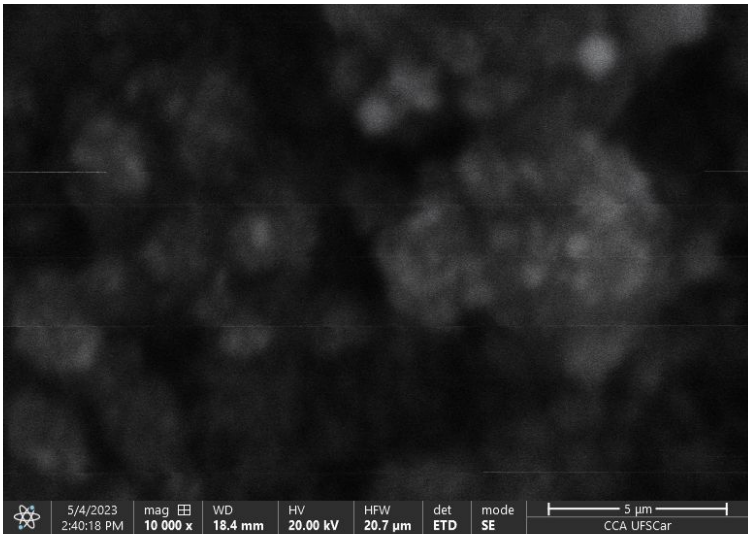

Map 1

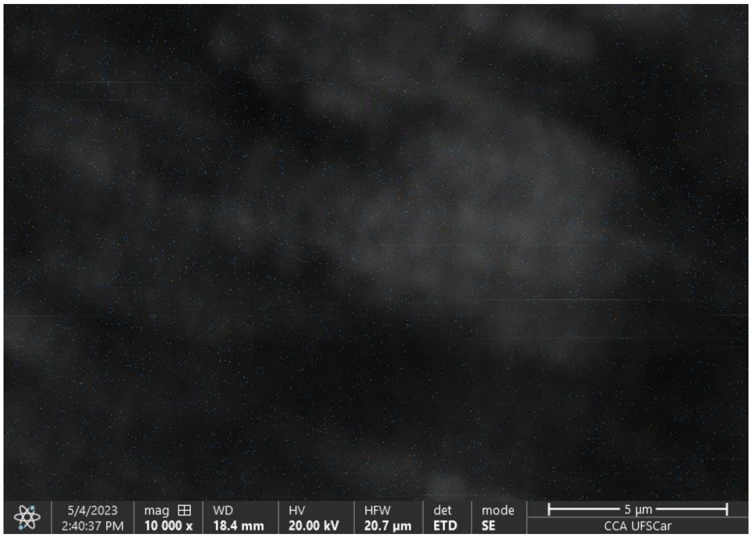

Total Number of Counts: 1459
Total Acquisition Time: 9 seconds
Average Count Rate: 162 cps
Acceleration Voltage: 20 kV
Map Resolution: 768 x 512

Element	Atomic %	Atomic % Error	Weight %	Weight % Error
C	71.4	1.0	60.8	0.9
O	27.5	1.4	31.3	1.6
Pd	1.1	0.1	7.9	0.8

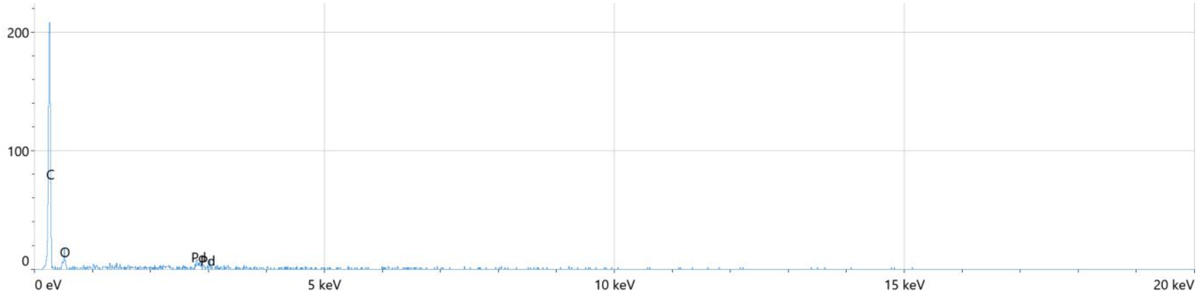

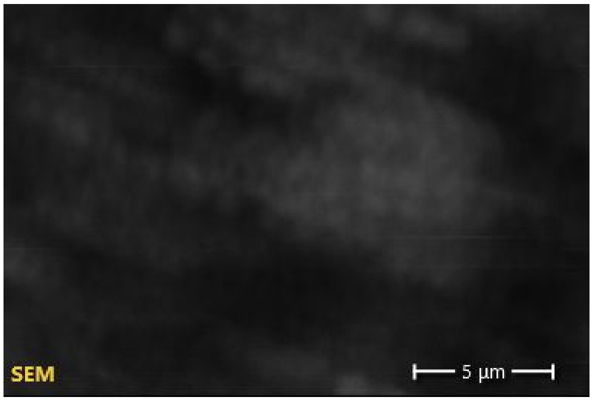

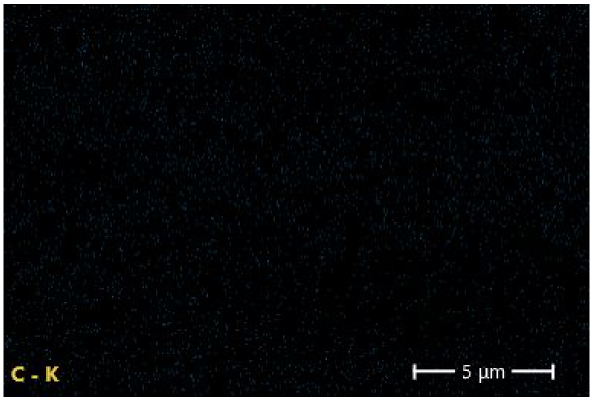

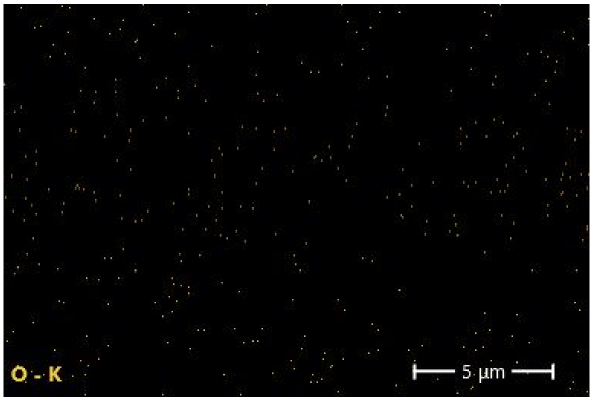
O - K
5 µm

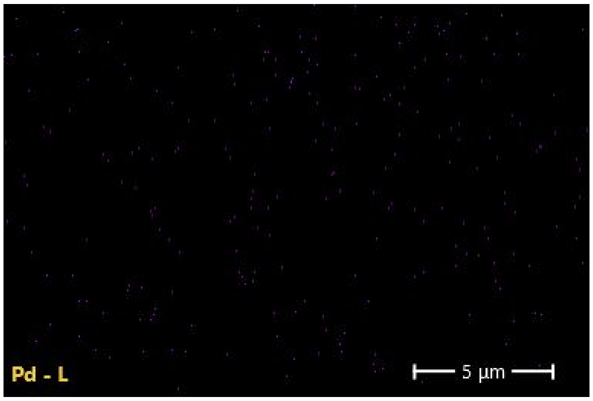
Pd - L
5 µm

Figure S9 - EDS performed on the Pd-CNTs-GV/ePAD sensor after 10-minutes electrodeposition of Pd solution of 1.0 mmol L^{-1} in HNO3 0.1 mol L^{-1} by chronoamperometry (potential applied: –0.7 V; interval time: 0.01s)

Session 23-05-04 14:20

Site 2

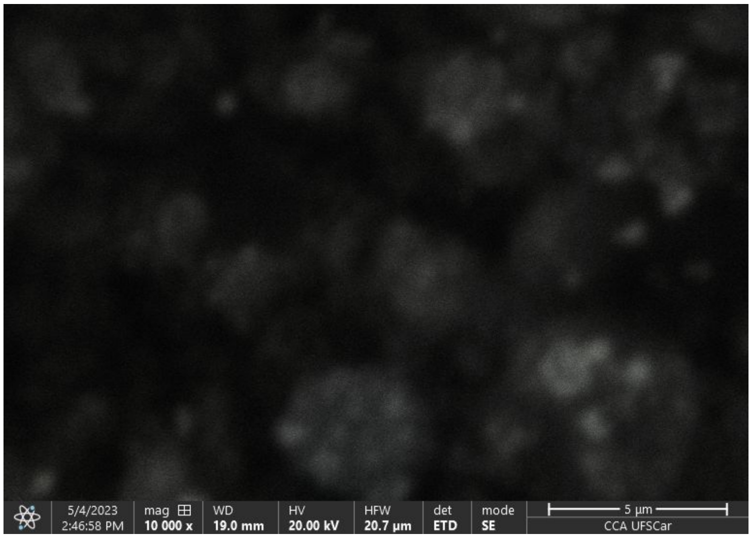

Map 2

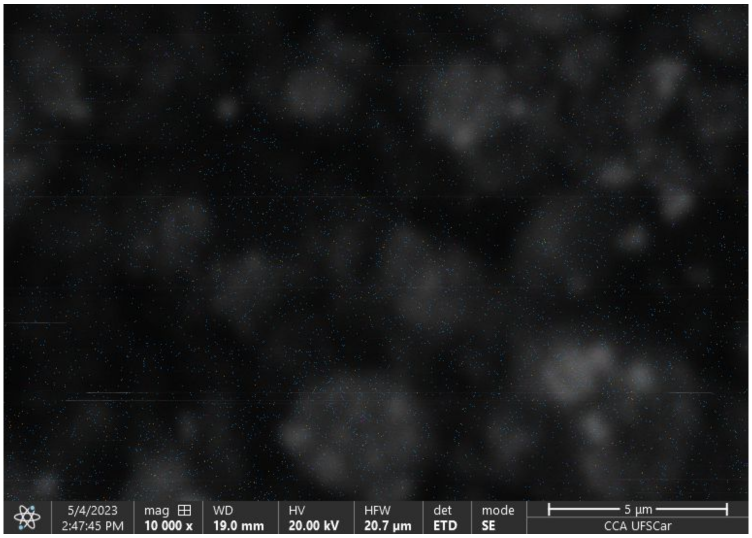

Total Number of Counts: 1576
Total Acquisition Time: 9 seconds
Average Count Rate: 175 cps
Acceleration Voltage: 20 kV
Map Resolution: 768 x 512

Element	Atomic %	Atomic % Error	Weight %	Weight % Error
C	84.8	0.9	74.8	0.8
O	14.1	1.4	16.6	1.7
Pd	1.1	0.1	8.6	0.9

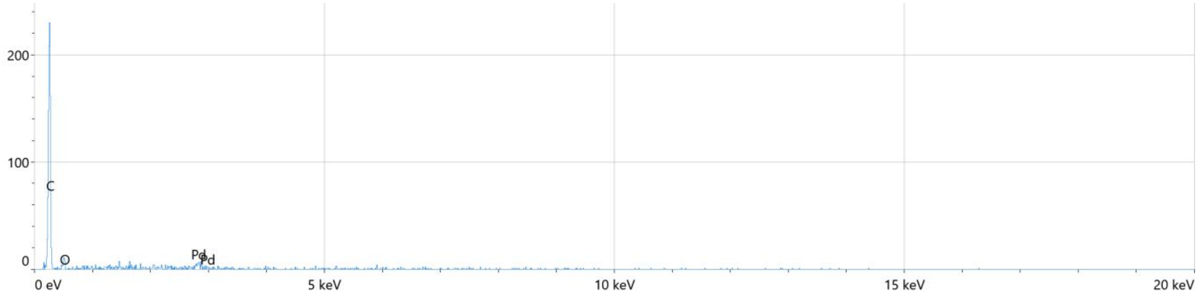

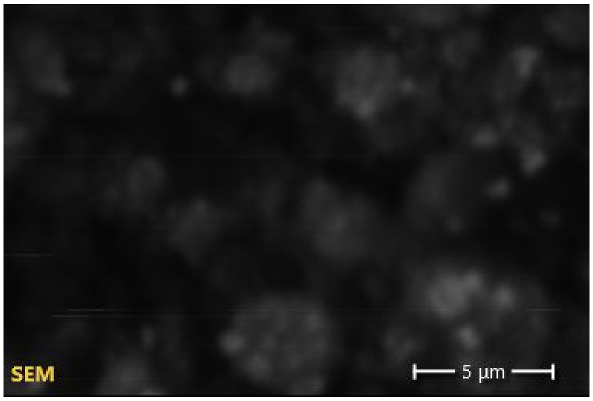

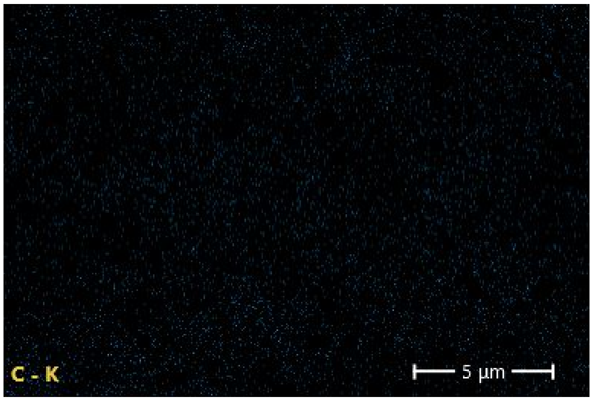

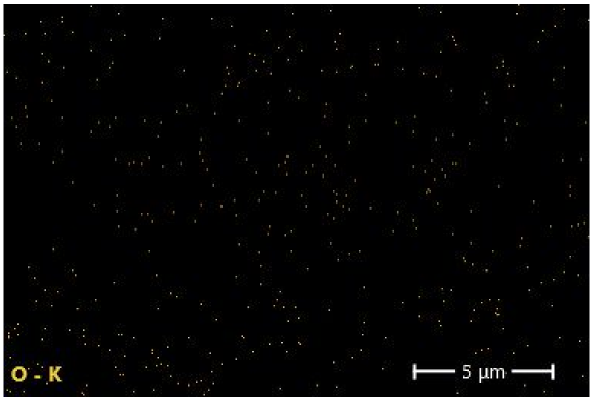
O - K
5 µm

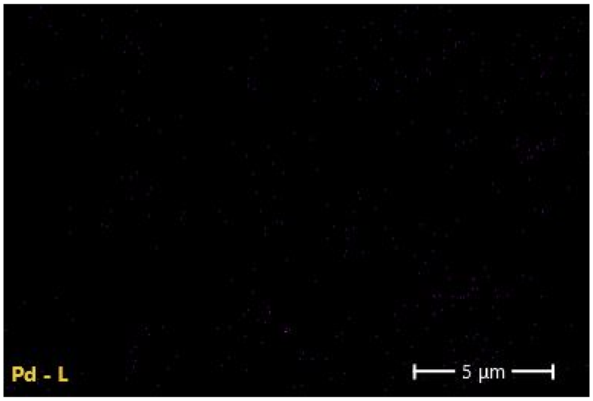
Pd - L
5 µm

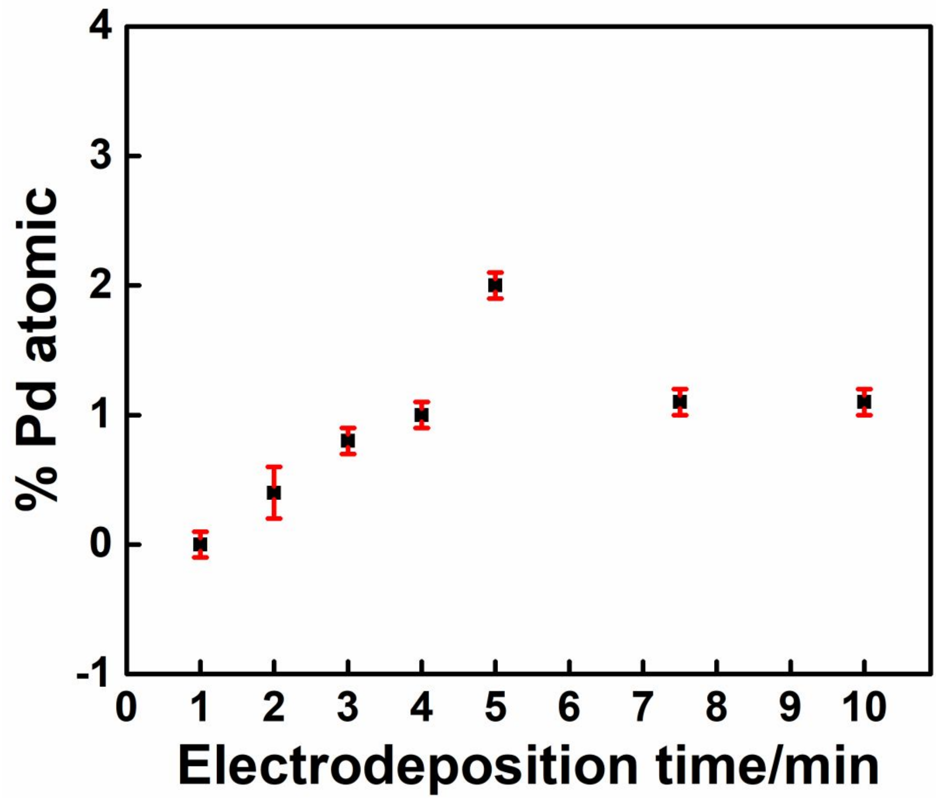

Figure S10. Percentage of atomic Pd after the electrodeposition time on the working electrode surface at different times. Data obtained by EDS analyses.

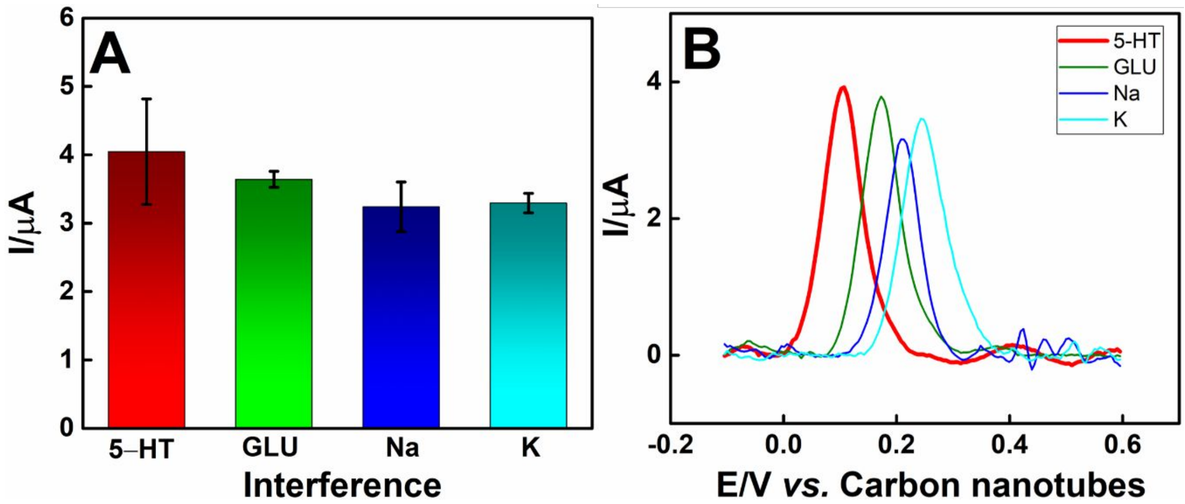

Figure S11. Interference studies. (A) Bar graph comparing the current obtained by the Pd−CNTs−GV/ePAD sensor in the presence of 100 μmol.L^{-1} of 5−HT in 0.1 mol.L^{-1} PB in the presence of 0.1 mol.L^{-1} KCl and 0.1 mol.L^{-1} NaCl (red); 100 μmol.L^{-1} of 5−HT and 100 μmol.L^{-1} of glucose in 0.1 mol.L^{-1} PB in the presence of 0.1 mol.L^{-1} KCl and 0.1 mol.L^{-1} NaCl (green); 100 μmol.L^{-1} of 5−HT in 0.1 mol.L^{-1} PB in the presence of 0.1 mol.L^{-1} KCL and 1.0 mol.L^{-1} NaCl (blue), and 100 μmol.L^{-1} of 5−HT in 0.1 mol.L^{-1} PB in the presence of 1.0 mol.L^{-1} KCl and 0.1 mol.L^{-1} NaCl (cyan). (B) DPV of the interferers.

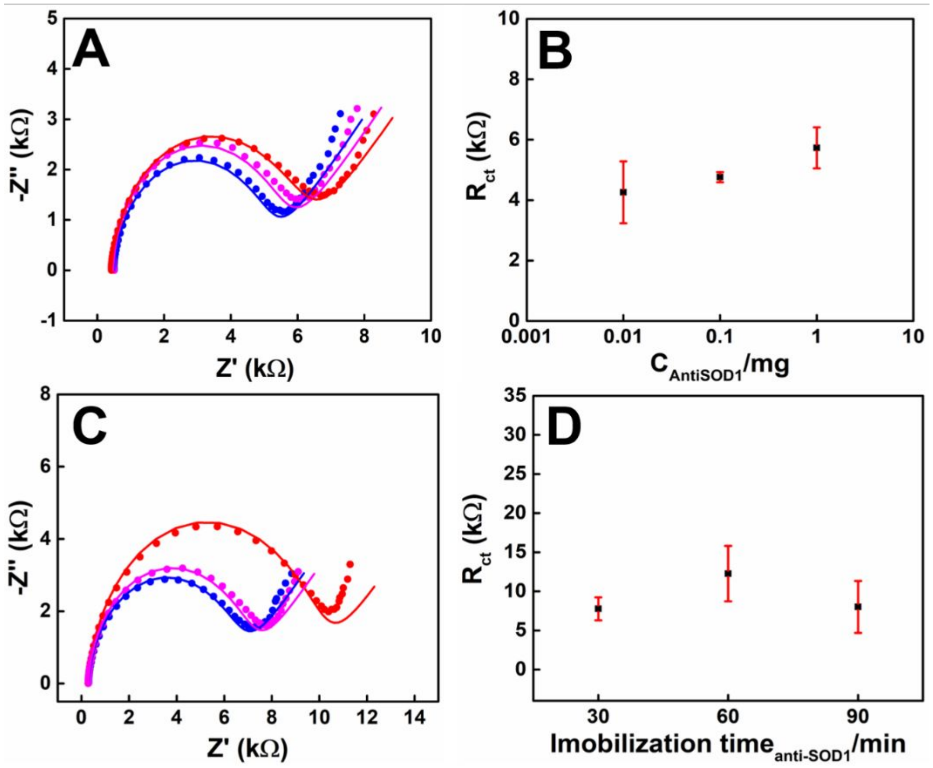

Figure S12. Study of the AntiSOD1 parameters for the construction of the electrochemical immunosensor. (A) Nyquist diagrams obtained after the immobilization (for 30 minutes) of 0.01 (blue), 0.10 (pink), and 1.0 mg (red) of the AntiSOD1 on the working electrode surface in the presence of $[Fe(CN)_6]^{4-/3-}$ in 0.1 mol L^{-1} KCl; The diagrams were obtained utilizing the $E_{1/2}$ = 0 V; data presented are normalized on both axes. (B) Graphic dot of AntiSOD1 concentration. (C) Nyquist diagrams obtained after the immobilization of 1.0 mg of the AntiSOD1 for 30 (blue), 60 (red), and 90 minutes (pink) in the presence of $[Fe(CN)_6]^{4-/3-}$ in 0.1 mol L^{-1} KCl; The diagrams were obtained utilizing the $E_{1/2}$ = 0 V; data presented are normalized on both axes. (D) Graphic dot of AntiSOD1 immobilization time.

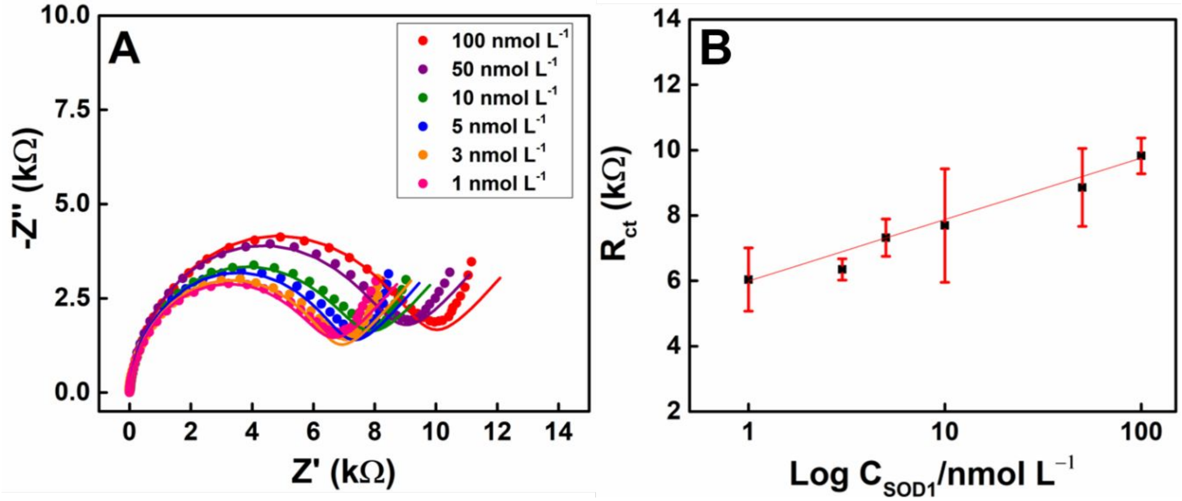

Figure S13. (A) Nyquist diagrams obtained by modified SOD1-BSA-AntiSOD1-GA-CYS-Pd/CNTs-GV/paper with different concentrations of SOD1 in 0.1 mol L^{-1} PBS, pH=7.4 (1.00, 3.00, 5.00, 10.0, 50.0, and 100 nmol L^{-1}), in the presence of $[Fe(CN)_6]^{4-/3-}$ in 0.1 mol L^{-1} KCl, E = 0V, data presented are normalized on both axes. (B) R_{ct} vs C_{SOD1} correlation

Table S1. Comparison between the linear range and LOD to 5-HT with literature works

Devices	Linear range ($\mu mol\ L^{-1}$)	LOD ($\mu mol\ L^{-1}$)	Works
**ZMCPE**	10.0 a 50.0	0.585	[36]
**FeC-AuNPs-MWCNTs**	0.05 a 20.0	0.017	[37]
**CDP-Choline/MCPE**	10.0 a 30.0	5.81	[38]
**Gr-AV**	6.00 a 100	0.390	[39]
**3D/rGO-PLA**	0.30 a 10.0	0.032	[40]
**Pd-CNTs-GV/ePAD**	**7.00 a 100**	**0.35**	**This work**

ZMCPE: electrode based on carbon paste modified with zirconia oxide nanoparticles; **FeC–AuNPs–MWCNTs:** screen-printed sensor using gold nanoparticles covalently bonded to ferrocene in carbon nanotubes; **CDP–Choline/MCPE:** carbon paste electrode with sodium citicoline; **Gr-AV:** disposable electrochemical sensor based in conductive ink of automotive varnish and graphite; **3D/rGO–PLA:** reduced graphene oxide and polylactic acid for 3D electrodes; **Pd-CNTs-GV/ePAD:** electrochemical sensor based in conductive ink with palladium electrodepositing on paper substrate

Table S2. 5−HT determination in the Pd-CNTs-GV/ePAD sensor in diluted human serum (1:100 (v:v) in 0.1 mol L^{-1} PBS (pH 7.5)

**Sample**	**Serotonin concentration (μmol L^{-1})**			
	**Added**	**Recovered**	**Recovery (%)**	**Error (%)**
**Diluted human serum (1:100 v:v PBS)**	100	96.2	96.2	± 4.5
	50.0	51.1	102.3	± 1.9
	7.00	7.56	108.0	± 1.8

Table S3. Approximate value of sensor preparation components

Components	Market value (Dollar)	Value for the production of 2.3 g of ink (Dollar)	
Glass Varnish (ACRILEX®) 100 mL	5.3446	0.107 (2 g)	
Carbon nanotubes 200g	483.00	0.7245 (0.3 g)	
Beeswax 150g	6.519	0.04346 (1g per sheet)	
A4 blue screen paper (Ref: 66668793 180g/m^2) 15 sheets	4.3952	0.21976 (1 sheet)	
Palladium(II) nitrate hydrate 2g	365.5063	0.42 (2.3mg in 10 mL to obtain 1.0 mmol $L^{-1)}$	
**Total**	-	**~1.51 dollar/20 units**	**0.076 dollar/unit**
